# Evaluation of Autoantibody Binding to Cardiac Tissue in Multisystem Inflammatory Syndrome in Children and COVID-19 Vaccination–Induced Myocarditis

**DOI:** 10.1001/jamanetworkopen.2023.14291

**Published:** 2023-05-18

**Authors:** Harsita Patel, Amalia Sintou, Rasheda A. Chowdhury, Stephen Rothery, Alma Octavia Iacob, Sanjay Prasad, Peter P. Rainer, Federico Martinón-Torres, Vanessa Sancho-Shimizu, Chisato Shimizu, Kirsten Dummer, Adriana H. Tremoulet, Jane C. Burns, Susanne Sattler, Michael Levin

**Affiliations:** 1 Department of Infectious Disease, Section of Paediatric Infectious Disease, Imperial College London, United Kingdom; 2 National Heart & Lung Institute, Faculty of Medicine, Imperial College London, United Kingdom; 3 Royal Brompton & Harefield hospitals, Guy’s and St Thomas’ National Health Service Foundation Trust, United Kingdom; 4 Department of Cardiology, Medical University of Graz, Graz, Austria; 5 BioTechMed Graz, Graz, Austria; 6 Genetics, Vaccines and Infections Research Group (GENVIP), Instituto de Investigación Sanitaria de Santiago, Santiago de Compostela, Spain; 7 Centro de Investigación Biomédica en Red de Enfermedades Respiratorias (CIBERES), Instituto de Salud Carlos III, Madrid, Spain; 8 Translational Pediatrics and Infectious Diseases, Department of Pediatrics, Hospital Clínico Universitario de Santiago de Compostela, Santiago de Compostela, Spain; 9 Department of Infectious Disease, Section of Virology, Imperial College London, London, United Kingdom; 10 Centre for Paediatrics and Child Health, Faculty of Medicine, Imperial College London, London, United Kingdom; 11 Department of Pediatrics, School of Medicine, University of California, San Diego, San Diego; 12 Rady Children’s Hospital, San Diego, California

## Abstract

**Importance:**

Cardiac dysfunction and myocarditis have emerged as serious complications of multisystem inflammatory syndrome in children (MIS-C) and vaccines against SARS-CoV-2. Understanding the role of autoantibodies in these conditions is essential for guiding MIS-C management and vaccination strategies in children.

**Objective:**

To investigate the presence of anticardiac autoantibodies in MIS-C or COVID-19 vaccine-induced myocarditis.

**Design, Setting, and Participants:**

This diagnostic study included children with acute MIS-C or acute vaccine myocarditis, adults with myocarditis or inflammatory cardiomyopathy, healthy children prior to the COVID-19 pandemic, and healthy COVID-19 vaccinated adults. Participants were recruited into research studies in the US, United Kingdom, and Austria starting January 2021. Immunoglobulin G (IgG), IgM, and IgA anticardiac autoantibodies were identified with immunofluorescence staining of left ventricular myocardial tissue from 2 human donors treated with sera from patients and controls. Secondary antibodies were fluorescein isothiocyanate–conjugated antihuman IgG, IgM, and IgA. Images were taken for detection of specific IgG, IgM, and IgA deposits and measurement of fluorescein isothiocyanate fluorescence intensity. Data were analyzed through March 10, 2023.

**Main Outcomes and Measures:**

IgG, IgM and IgA antibody binding to cardiac tissue.

**Results:**

By cohort, there were a total of 10 children with MIS-C (median [IQR] age, 10 [13-14] years; 6 male), 10 with vaccine myocarditis (median age, 15 [14-16] years; 10 male), 8 adults with myocarditis or inflammatory cardiomyopathy (median age, 55 [46-63] years; 6 male), 10 healthy pediatric controls (median age, 8 [13-14] years; 5 male), and 10 healthy vaccinated adults (all older than 21 years, 5 male). No antibody binding above background was observed in human cardiac tissue treated with sera from pediatric patients with MIS-C or vaccine myocarditis. One of the 8 adult patients with myocarditis or cardiomyopathy had positive IgG staining with raised fluorescence intensity (median [IQR] intensity, 11 060 [10 223-11 858] AU). There were no significant differences in median fluorescence intensity in all other patient cohorts compared with controls for IgG (MIS-C, 6033 [5834-6756] AU; vaccine myocarditis, 6392 [5710-6836] AU; adult myocarditis or inflammatory cardiomyopathy, 5688 [5277-5990] AU; healthy pediatric controls, 6235 [5924-6708] AU; healthy vaccinated adults, 7000 [6423-7739] AU), IgM (MIS-C, 3354 [3110-4043] AU; vaccine myocarditis, 3843 [3288-4748] AU; healthy pediatric controls, 3436 [3313-4237] AU; healthy vaccinated adults, 3543 [2997-4607] AU) and IgA (MIS-C, 3559 [2788-4466] AU; vaccine myocarditis, 4389 [2393-4780] AU; healthy pediatric controls, 3436 [2425-4077] AU; healthy vaccinated adults, 4561 [3164-6309] AU).

**Conclusions and Relevance:**

This etiological diagnostic study found no evidence of antibodies from MIS-C and COVID-19 vaccine myocarditis serum binding cardiac tissue, suggesting that the cardiac pathology in both conditions is unlikely to be driven by direct anticardiac antibody–mediated mechanisms.

## Key Points

**Question:**

Do autoantibodies targeting the heart play a role in the cardiac complications of SARS-CoV-2–associated multisystem inflammatory syndrome in children (MIS-C) or COVID-19 mRNA vaccination?

**Findings:**

This diagnostic study including 20 children with MIS-C or COVID 19 vaccine–induced myocarditis and 21 adult and pediatric controls found no evidence of autoantibodies in serum of patients with MIS-C or vaccine-induced myocarditis binding donor cardiac tissue.

**Meaning:**

These results suggest that anticardiac autoantibodies are unlikely to play a role in the cardiac pathology seen in MIS-C or COVID-19 vaccine–induced myocarditis.

## Teaser

This diagnostic study assesses the presence of anticardiac autoantibodies in children with multisystem inflammatory syndrome and COVID-19 vaccine–induced myocarditis.

## Introduction

As the COVID-19 pandemic progressed, new childhood disorders associated with SARS-CoV-2 emerged, including multisystem inflammatory syndrome in children (MIS-C) and COVID-19 vaccine-induced myocarditis. MIS-C typically occurs 4 to 6 weeks following exposure to SARS-CoV-2 in school-aged children (mean age, 9.3 years),^1^ with a higher prevalence in males and Black and Hispanic children.^2^ Children usually present with fever, rash, conjunctival injection and gastrointestinal symptoms.^1^ A significant proportion (80%) have cardiac involvement that is associated with elevated cardiac troponin and brain natriuretic peptide.^3^ Severe cases develop impaired cardiac function, shock, and multisystem failure requiring inotropic support and intensive care. Echocardiograms most frequently show left ventricular dysfunction and coronary artery dilatation or aneurysms, and arrythmias may be detected on electrocardiography.^3^ Despite the severe acute illness, data from a 6-month follow-up study are reassuring, with nearly all patients returning to baseline cardiac function between 2 and 6 months.^4^ Considering that MIS-C occurs several weeks following exposure to SARS-CoV-2, it is possible that a dysregulated adaptive immune response (mediated by autoantibodies or T cells) is driving the disease process and cardiac dysfunction and failure.^5,6^

Vaccine-induced myocarditis is a rare complication following mRNA-based COVID-19 vaccination, which is most prevalent in males aged 12 to 24 years, with an estimated rate of 52.4 to 105.9 cases per million doses.^7,8^ Patients typically present 1 to 3 days following the second dose with chest pain and elevated cardiac troponin measures. Serology and polymerase chase reaction tests are negative for common causes of viral myocarditis.^8^ Typically, ST elevation is seen on electrocardiograms, as well as left ventricular dysfunction on echocardiograms and, weeks after the acute presentation, late gadolinium enhancement (representing myocardial fibrosis) on cardiac magnetic resonance imaging.^8,9^ COVID-19 vaccine-induced myocarditis appears to be self-limiting, and most patients require only supportive treatment (usually nonsteroidal anti-inflammatory agents).^9^ Several mechanisms of disease causation have been proposed including hormonal differences, delayed hypersensitivity reaction, microvessel thrombosis leading to ischemia, genetic susceptibility, and the generation of antibodies directed against spike protein epitopes that cross-react with myocardial tissues.^10,11,12^

The mechanisms underlying cardiac pathology in MIS-C and COVID-19 vaccine-induced myocarditis have yet to be elucidated, with limited experimental data published to date. The adaptive immune response may play a role in both conditions. We therefore investigated the presence of autoantibodies targeting the heart as a potential mechanism for the cardiac involvement in both MIS-C and COVID-19 vaccine–induced myocarditis.

## Methods

### Study Design

This ex vivo diagnostic study investigated the presence of immunoglobulin G (IgG), immunoglobulin M (IgM), and immunoglobulin A (IgA) antibodies by immunofluorescence staining of left ventricular cardiac tissue from 2 human adult donors treated with sera from patients and controls. Patients with MIS-C and healthy vaccinated adults were recruited for this study, while serum samples from vaccine myocarditis patients, patients with adult myocarditis or inflammatory cardiomyopathy, and healthy prepandemic pediatric controls were obtained from existing bioregisteries where patients had consented for use of samples in future studies. This study began January 8, 2021, and is scheduled to conclude October 3, 2023. This report follows the Transparent Reporting of a Multivariable Prediction Model for Individual Prognosis or Diagnosis (TRIPOD) reporting guidelines.

### Clinical Cohorts

Children with MIS-C and adult healthy controls were recruited as part of the multicenter European Union–funded Diagnosis and Management of Febrile Illness using RNA Personalised Molecular Signature Diagnosis Study (DIAMONDS). Ethical approval was obtained from the UK research ethics committee with written informed consent obtained from all participants. Prepandemic COVID-19 healthy pediatric controls were from the European Union Childhood Life-threatening Infectious Disease Study (EUCLIDS); ethical approval was obtained from the UK research ethics committee with written informed consent obtained for samples to be used in future studies. COVID-19 vaccine-induced myocarditis patients were recruited from Rady Children’s Hospital San Diego as part of the Diagnosing and Predicting Risk in Children with SARS-CoV-2–Related Illness study. The study was approved by the University of California, San Diego institutional review board, and parents and patients signed informed consent and assent documents as appropriate. Adult myocarditis and inflammatory cardiomyopathy patients were recruited in 2 separate studies following written informed consent in the Diagnosis and Risk Stratification in Myocarditis study. Ethical approval was obtained from the UK research ethics committee and the Medical University of Graz institutional review board.

Myocardial tissue from organ donors was obtained as part of the Structure and Functional Characterization of the Human Heart Study, with written informed consent for research given by the patient’s family when all clinical usage was exhausted. Details of donors A and B, along with details of cardiac tissue collection and storage can be found in eTable 1 in Supplement 1. The UK research ethics committee approved the study.

### Data Collection and Definitions

This study used anonymized clinical data, including demographic information, collected from medical records as part of the DIAMONDS, EUCLIDS, Diagnosing and Predicting Risk in Children with SARS-CoV-2– Related Illness, Structure and Functional Characterization of the Human Heart, and the Graz Endomyocardial Biopsy Registry studies. Self-reported ethnicity data has been included in this study as ethnic disparities have been widely reported in SARS-CoV-2 associated disease; categories included Arab, Black, Caucasian, Hispanic, South Asian, mixed, and unknown.

### Inclusion and Exclusion Criteria

Eligible participants were children and young adults (aged under 19 years) diagnosed with MIS-C who were admitted to hospital with an acute inflammatory febrile illness meeting the World Health Organization case definition of MIS-C.^13^ Patients with MIS-C who had cardiac involvement requiring intensive care were included, and patients with MIS-C with no cardiac involvement (defined by no significant abnormalities in cardiac troponin and echocardiogram findings) were recruited from the ward. All MIS-C serum samples were obtained within 72 hours of admission to hospital, during the acute inflammatory phase of their illness and prior to receiving any immunomodulatory treatment. Patients with other concurrent infectious disease were excluded.

The vaccinated myocarditis cohort included children and young adults presenting with symptoms of myocarditis following second dose of mRNA vaccination, where other causes of myocarditis had been excluded. Serum samples were taken on the day of admission to hospital prior to receiving any treatment. The adult myocarditis and inflammatory cardiomyopathy cohort excluded patients with infectious causes of myocarditis.

For the healthy controls, healthy children (aged under 19 years) were recruited prior to the COVID-19 pandemic. Healthy COVID-19–vaccinated adults were aged over 21 years and had blood drawn between 2 and 12 weeks following vaccination. Cardiac donors were excluded if they had preexisting cardiovascular disease, autoimmune conditions, or prolonged ischemia before tissue was harvested from the left ventricle (eTable 1 in Supplement 1).

### Experimental Procedure

Immunohistochemistry was performed on tissue sections from the left ventricular apex from 2 donor hearts for assessment of autoantibody binding. Sera from patients and controls were used as primary antibodies. Fluorescein isothiocyanate (FITC)-conjugated antihuman IgG, IgM, and IgA (Agilent Technologies) were used for autoantibody detection. Serum from one of the adult myocarditis or inflammatory myocarditis case was used as a positive experimental control. Ten representative images were taken at random from each section on a widefield microscope (HWF1 Zeiss Axio Observer). Immunoglobulin deposition was assessed qualitatively and quantified by calculating fluorescence intensity using ImageJ/Fiji software.^14,15^

### Outcome Measures

The primary qualitative outcome was the presence of specific IgG, IgM, and IgA binding to cardiac tissue. Fluorescence intensity of IgG, IgM, and IgA staining was measured.

### Statistical Analysis

Prism version 8.4.3 (GraphPad) was used for data analysis. Kruskal-Wallis test was performed to compare median fluorescence intensity of IgG, IgM, and IgA staining between clinical cohorts. The threshold for statistical significance was *P* < .05.

## Results

### Clinical Characteristics

By cohort, a total of 10 children with MIS-C (median [IQR] age, 10 [13-14] years), 10 children with vaccine myocarditis (median [IQR] age, 15 [14-16] years), 8 adults with myocarditis or inflammatory cardiomyopathy (median [IQR] age, 55 [46-63] years), 10 healthy pediatric controls (median [IQR] age, 8 [13-14] years), and 10 healthy vaccinated adults (all older than 21 years) were included (Table; eTable 2 in Supplement 1). There was an equal sex distribution in the healthy pediatric and healthy vaccinated adult groups, while there was a preponderance of male participants in the MIS-C (6 of 10 male), adult myocarditis and inflammatory cardiomyopathy (6 of 8 male), and vaccine myocarditis (10 of 10 male) groups, in keeping with the reported male predominance in these conditions. Ethnic minority groups were overrepresented in MIS-C, but this trend was not observed in the other cohorts. The adult non–SARS-CoV-2 myocarditis (positive control) sample was from a 41-year-old South Asian man with a diagnosis of acute lymphocytic myocarditis. The clinical characteristics of the remaining 7 adult myocarditis and inflammatory cardiomyopathy patients can be found in eTable 2 in Supplement 1.

**Table.** Demographic and Clinical Characteristics of Patients and Controls

Characteristics	Participants, No. (%)				
	Vaccine myocarditis (n = 10)	MIS-C (n = 10)	Healthy pre-pandemic pediatric controls (n = 10)	Healthy vaccinated adults (n = 10)	Adult myocarditis (n = 1)
Age, median (IQR), y	15 (14-16)	10 (13-14)	8 (12.5-14)	>21	41
Male, No. (%)	10 (100)	6 (60)	5 (50)	5 (50)	1 (100)
Ethnicity, No. (%)					
Arab	0	0	2 (20)	0	NA
Black	0	2 (20)	2 (20)	1 (10)	NA
Caucasian	5 (50)	2 (20)	3 (30)	7 (70)	NA
Hispanic	4 (40)	0	0	1 (10)	NA
South Asian	0	4 (40)	2 (20)	1 (10)	1 (100)
Mixed	1 (10)	1 (10)	0	0	NA
Unknown	0	1 (10)	0	0	NA
Comorbidities, No. (%)					
ADHD	1 (10)	0	NR	NR	NR
Asthma	0	1 (10)	NR	NR	NR
Autism	1 (10)	0	NR	NR	NR
Developmental delay	1 (10)	0	NR	NR	NR
Eczema	0	2 (20)	NR	NR	NR
Myelomeningocele	1 (10)	0	NR	NR	NR
Obesity	2 (20)	1 (10)	NR	NR	NR
Maximum cardiac troponin, median (IQR), ng/L^a^	7355 (2562.5-17262.5)	86 (26-384)	NR	NR	2596
Maximum C-reactive protein, median (IQR), mg/L^b^	34 (16-37.5)^c^	227 (189-293)	NR	NR	89
Echocardiogram findings					
Coronary artery z-score >2.5	0	2 (20)	NR	NR	0
Left ventricular ejection fraction <55%	5 (50)	3 (30)	NR	NR	1 (100)
Right ventricular dysfunction	1 (10)	0	NR	NR	0
Valvular regurgitation^d^	1 (10)	1 (10)	NR	NR	0
Cardiac MRI findings					
T1 hyperenhancement	5 (50)	NR	NR	NR	NR
T2 hyperenhancement	4 (40)	NR	NR	NR	NR
Postcontrast delayed enhancement	3 (30)	NR	NR	NR	NR
Shock, No. (%)	0	6 (60)	NA	NA	1 (100)
Inotrope requirement, No. (%)	0	6 (60)	NA	NA	1 (100)
Intensive care support, No. (%)	0	5 (50)	NA	NA	1 (100)
SARS-CoV-2 PCR positive, No. (%)	0	0	NR	NR	0
SARS-CoV-2 spike IgG, median (IQR)^e^	76.9 (73.6-94.4)^f^	4.7 (4.7-4.7)^g^	0.27 (0.34-0.46)	1.9 (2.3-3.0)	NR
SARS-CoV-2 nucleocapsid IgG positive	1 (10)	NR	NR	NR	NR
Immunomodulatory treatment	10 (100)^h^	7 (70)^i^	NA	NA	0

Abbreviations: ADHD, attention-deficit/hyperactivity disorder; IgG, immunoglobulin G; NA, not applicable; NR, not reported; PCR, polymerase chain reaction.

To convert troponin to micrograms per liter, multiply by 1.0.

^a^ Normal range is below 14 ng/L.

^b^ Normal range is below 3 mg/L.

^c^ Performed on 7 of 10 patients.

^d^ Valvular regurgitation was defined as anything more severe than mild tricuspid or mild mitral valve regurgitation.

^e^ Normal range is between 0 and 2.

^f^ Performed on 3 of 10 patients.

^g^ Performed on 7 of 10 patients.

^h^ All 10 participants treated with ibuprofen and 1 participant (10%) concurrently with prednisolone.

^i^ Treatment included 6 participants (60%) with glucocorticoids, 5 (50%) with intravenous immunoglobulin, 3 (30%) with tocilizumab, and 1 (10%) with anakinra.

### Immunohistochemistry

#### IgG Staining

**Figure 1.**
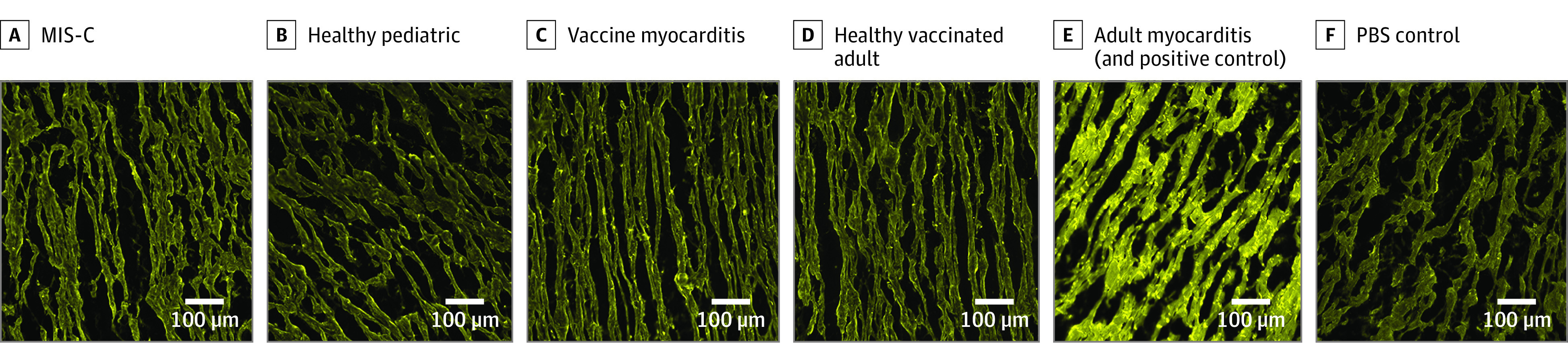
Immunohistochemistry Images of Cardiac Tissue From Donor A Treated With Serum From Patients and Controls and Stained With Fluorescein Isothiocyanate (FIT-C)–Conjugated Antihuman Immunoglobulin G Images were taken on wide-field microscope using a 20× (0.8 NA) objective. Cardiac tissue was treated with serum diluted at 1:50. MIS-C indicates multisystem inflammatory syndrome in children; PBS, phosphate-buffered saline.

**Figure 2.**
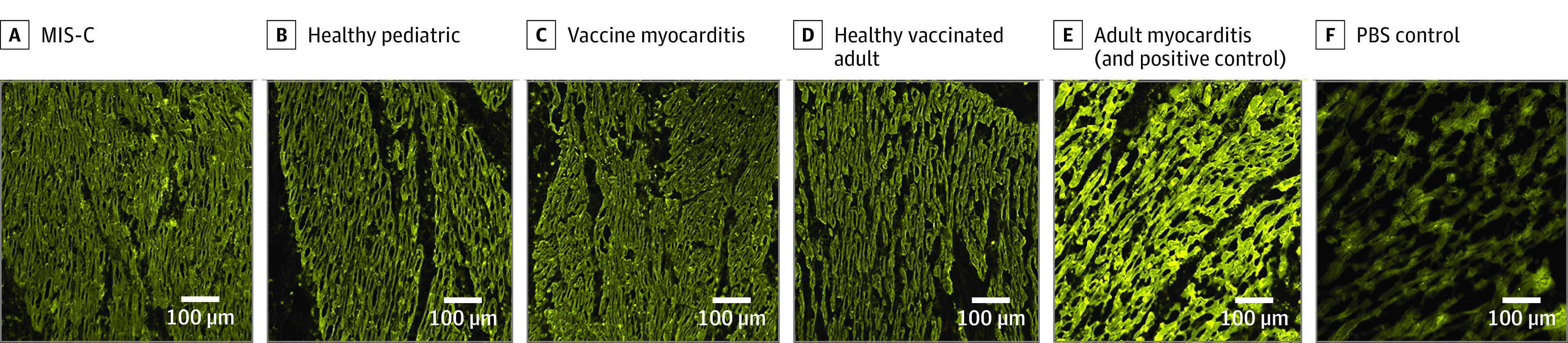
Immunohistochemistry Images of Cardiac Tissue From Donor B Treated With Serum From Patients and Controls and Stained With Fluorescein Isothiocyanate (FIT-C)–Conjugated Antihuman Immunoglobulin G Images were taken on wide-field microscope using a 20× (0.8 NA) objective. Cardiac tissue was treated with serum diluted at 1:50. MIS-C indicates multisystem inflammatory syndrome in children; PBS, phosphate-buffered saline.

IgG staining was performed using serum from all study participants. No specific binding was observed in left ventricular tissue treated with sera from patients with MIS-C, COVID-19 vaccine–induced myocarditis, and 7 of 8 patients in the acute myocarditis and inflammatory cardiomyopathy cohort. Figure 1 shows the staining obtained using serum from 1 of the adult myocarditis patients (positive control), with specific binding of IgG to myocardial structures in cardiac tissue. This finding was reproducible on 3 separate experiments in cardiac tissue from both donors A and B (eFigure 1 in Supplement 1). In comparison, sera from COVID-19 vaccine–induced myocarditis and patients with MIS-C, as well as 7 of 8 adults with myocarditis and inflammatory cardiomyopathy did not show any staining for IgG (Figure 1; eFigure 6 in Supplement 1). Quantified fluorescence intensity signal of FITC IgG deposition in MIS-C, COVID-19 vaccine–induced myocarditis and 7 of 8 adults with myocarditis and inflammatory cardiomyopathy were similar to healthy controls (*P* > .05 for all Kruskal-Wallis comparisons), but markedly lower than the adult myocarditis positive control (Figure 2).

#### IgM and IgA Staining

**Figure 3.**
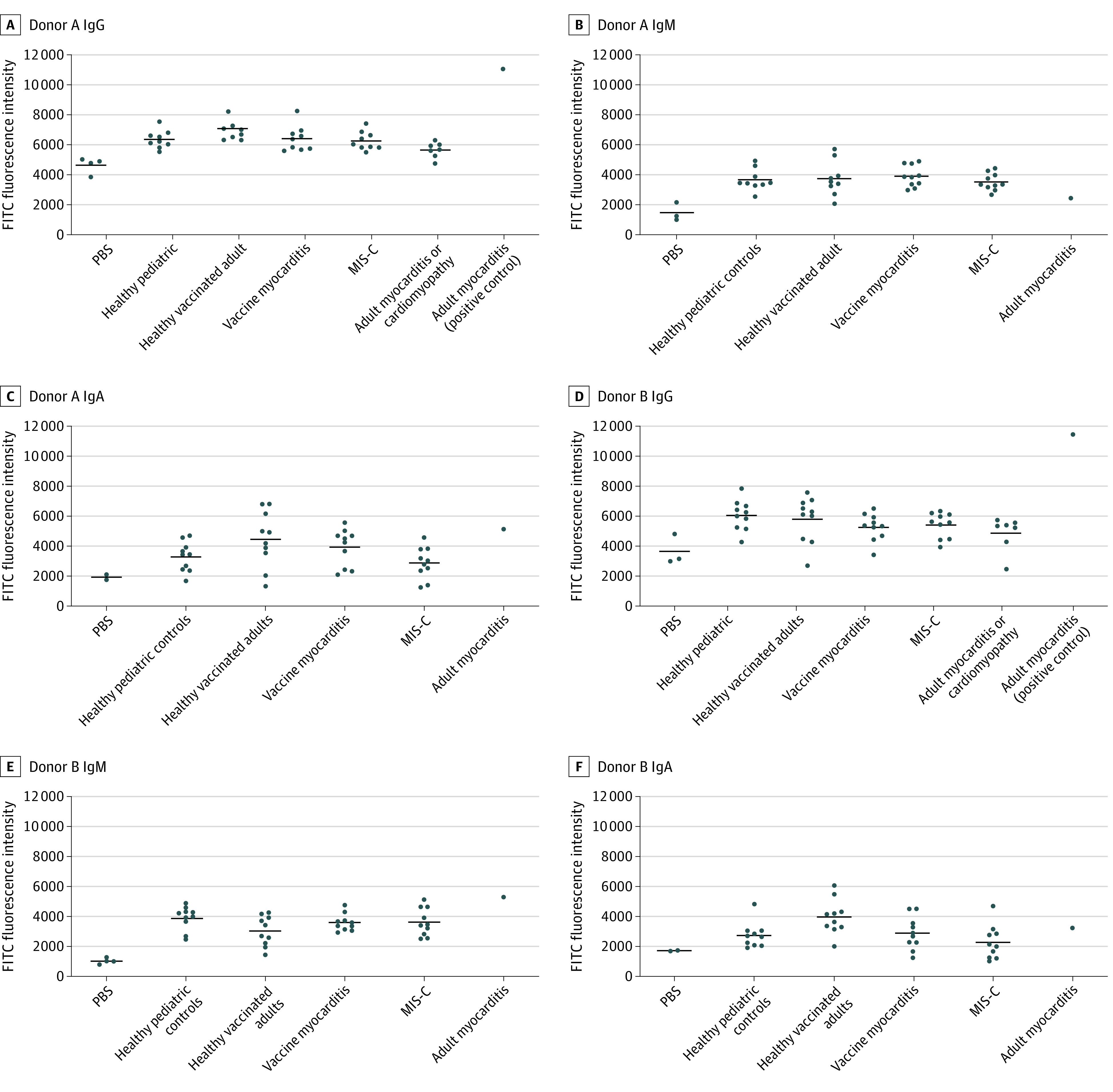
Fluorescence Intensity of Fluorescein Isothiocyanate (FITC) Conjugated With Antihuman Immunoglobulin G (IgG), IgM, and IgA in Cardiac Tissue Cohorts included 10 healthy prepandemic pediatric controls, 10 healthy COVID-19–vaccinated adults, 10 patients with COVID-19 vaccine myocarditis, 10 patients with multisystem inflammatory syndrome in children (MIS-C), 1 adult with myocarditis (positive control for immunoglobulin G [IgG]), 7 patients with adult myocarditis or inflammatory cardiomyopathy, and 2 to 4 phosphate buffered saline–negative controls. Only IgG measurements were taken for the adult myocarditis or inflammatory cardiomyopathy cases. No significant difference seen between MIS-C, vaccine myocarditis, adult myocarditis or inflammatory cardiomyopathy, and healthy pediatric and adult controls on Kruskal-Wallis analysis. PBS indicates phosphate-buffered saline.

IgM and IgA staining was performed using serum from patients with MIS-C, patients with COVID-19 vaccine–induced myocarditis, healthy pediatric controls, healthy COVID-19 vaccinated adults, and 1 adult myocarditis patient (IgG staining positive control) (Figure 3). No specific staining was seen for IgM and IgA for any patients or controls (eFigures 2 through 5 in Supplement 1). No significant differences were observed in the fluorescence intensity signal of FITC IgM and FITC IgA (Figure 1) when comparing patients and controls (*P* > .05 for all Kruskal-Wallis comparisons).

## Discussion

The findings of this study suggest that the cardiac injury in MIS-C and vaccine myocarditis is likely not to be solely antibody mediated or through direct anticardiac antibody–mediated mechanisms. Moreover, only 1 of 8 adults with myocarditis and inflammatory cardiomyopathy showed IgG deposition on immunohistochemistry, which is in keeping with previously reported immunofluorescence detection rates of cardiac autoantibodies in myocarditis and cardiomyopathy.^16^

Several studies have implicated antibodies in MIS-C^5,17,18^ and vaccine myocarditis^19,20,21^ immunopathology; however, functional evidence of the mechanism of cardiac dysfunction remains unclear. Alternatively, it is plausible that the presence of both viral antigens and antibodies may be required for a direct antibody-mediated cardiac pathology, which is supported by findings of 2 studies from Yonker et al showing viral antigenemia in patients with MIS-C^22^ and vaccine myocarditis.^23^ However, this is contrary to the findings of another study showing that most patients with MIS-C have undetectable levels of SARS-CoV-2 nucleocapsid and spike antigens in their blood.^24^

As the clinical profile, cardiac involvement, and disease severity of MIS-C and vaccine myocarditis are very different, these 2 conditions could potentially be caused by different dysregulated immune responses. MIS-C is associated with a systemic hyperinflammatory response and excessive cytokine release, which, along with rapid severe cardiac dysfunction that reverses quickly with immunomodulation, suggests that increased pro-inflammatory cytokines (eg, interlukin 6, interferon γ, tumor necrosis factor α) could be contributing to the cardiac dysfunction. The inflammatory response in vaccine myocarditis is comparatively less severe, and more organ specific. Combined with reports of eosinophilia in these cases,^25,26^ evidence supports the hypothesis of a delayed hypersensitivity response.

Given that MIS-C and vaccine myocarditis both occur several weeks following exposure to SARS-CoV-2 antigens, it is probable that an aberrant adaptive immune response is responsible. If this is indeed the case, it would be important to investigate T cell responses in both these conditions. Ultimately, it is likely that the etiology is multifactorial, whereby exposure to a novel antigen results in a dysregulated adaptive immune response in genetically susceptible individuals.

### Limitations

This study had several limitations. First, we only had 1 positive control to confirm the validity of the immunoglobulin detection methods. However, this single positive adult myocarditis control showed reproducible findings on 3 separate experiments, on cardiac tissue from 2 different donors. This implies that the presence of antibodies in human serum binding cardiac tissue can be reliably detected by this method. Second, MIS-C and vaccine myocarditis primarily affect children, while an adult positive control and adult cardiac donors have been used, which may not be truly representative. Third, it should be considered that the lack of antibody signal may be due to donor hearts not having the required genetic predisposition as those developing MIS-C cardiac complications, and vaccine myocarditis may have a different genetic background to the rest of the population. Fourth, while no differences in overall IgG, IgM, and IgA antibodies were seen, this study did not test for antibodies against specific antigens (eg, α-myosin).

## Conclusions

In this etiological study investigating the role of antibodies in MIS-C and COVID-19 vaccine myocarditis cardiac pathology, there was no evidence of antibodies from patient sera binding donor cardiac tissue. This suggests that direct anticardiac antibody–mediated mechanisms are unlikely to be driving the cardiac immunopathology in both these conditions and should stimulate further functional work to identify the mechanism of cardiac pathology in these 2 conditions.

## Notes

Supplement 1. **eTable 1.** Cardiac Donors and Details of Cardiac Tissue Collection and Storage **eTable 2.** Clinical and Demographic Details of Adult Myocarditis/Inflammatory Cardiomyopathy Cases **eFigure 1.** IgG Immunohistochemistry Images of Adult Myocarditis (Positive Control) Repeated on 3 Separate Experiments **eFigure 2.** IgM Immunohistochemistry Images—Donor A **eFigure 3.** IgM Immunohistochemistry Images—Donor B **eFigure 4.** IgA Immunohistochemistry Images—Donor A **eFigure 5.** IgA Immunohistochemistry Images—Donor B **eFigure 6.** IgG Immunohistochemistry Images for Adult Myocarditis/Inflammatory Cardiomyopathy Patients—Donor A

Supplement 2.
Nonauthor Collaborators

Supplement 3.
Data Sharing Statement
