# Supplementary material for: Evaluation of Autoantibody Binding to Cardiac Tissue in Multisystem Inflammatory Syndrome in Children and COVID-19 Vaccination–Induced Myocarditis
Source: JAMA Netw Open. 2023 May 18;6(5):e2314291. doi: 10.1001/jamanetworkopen.2023.14291 (PMC10196878; doi:10.1001/jamanetworkopen.2023.14291)
Supplement: Supplement 1. — eTable 1. Cardiac Donors and Details of Cardiac Tissue Collection and Storage eTable 2. Clinical and Demographic Details of Adult Myocarditis/Inflammatory Cardiomyopathy Cases eFigure 1. IgG Immunohistochemistry Images of Adult Myocarditis (Positive Control) Repeated on 3 Separate Experiments eFigure 2. IgM Immunohistochemistry Images—Donor A eFigure 3. IgM Immunohistochemistry Images—Donor B eFigure 4. IgA Immunohistochemistry Images—Donor A eFigure 5. IgA Immunohistochemistry Images—Donor B eFigure 6. IgG Immunohistochemistry Images for Adult Myocarditis/Inflammatory Cardiomyopathy Patients—Donor A [file jamanetwopen-e2314291-s001.pdf]

Supplemental Online Content

Patel H, Sintou A, Chowdhury RA, et al; DIAMONDS consortium. Evaluation of autoantibody binding to cardiac tissue in multisystem inflammatory syndrome in children and COVID-19 vaccination–induced myocarditis. *JAMA Netw Open.* 2023;6(5):e2314291. doi:10.1001/jamanetworkopen.2023.14291

eTable 1. Cardiac Donors and Details of Cardiac Tissue Collection and Storage

eTable 2. Clinical and Demographic Details of Adult Myocarditis/Inflammatory Cardiomyopathy Cases

eFigure 1. IgG Immunohistochemistry Images of Adult Myocarditis (Positive Control) Repeated on 3 Separate Experiments

eFigure 2. IgM Immunohistochemistry Images—Donor A

eFigure 3. IgM Immunohistochemistry Images—Donor B

eFigure 4. IgA Immunohistochemistry Images—Donor A

eFigure 5. IgA Immunohistochemistry Images—Donor B

eFigure 6. IgG Immunohistochemistry Images for Adult Myocarditis/Inflammatory Cardiomyopathy Patients—Donor A

This supplemental material has been provided by the authors to give readers additional information about their work.

© 2023 Patel H et al. *JAMA Network Open.*

eTable 1. Cardiac donors and details of cardiac tissue collection and storage

	Donor A	Donor B
**Age (years)**	48	17
**Sex**	Male	Female
**Past medical history**	Nil recorded	No history of: HTN/cardiothoracic disease/liver disease/diabetes/smoking/alcohol drinking.
**Cause of death**	Hemorrhage	Status Epilepticus
**Reason for unsuitability as transplant organ**	Poor function	Deterioration on organ care system
**Sample collection and storage**	The hearts were flash-frozen in liquid nitrogen, then stored at −80 °C for cryosectioning. Heart tissue was cut into 7μm thick sections using a cryostat (CM1850, Leica, Germany) and air-dried for 15 minutes before storing long-term at −80 °C freezer.	

© 2023 Patel H et al. *JAMA Network Open.*

eTable 2. Clinical and demographic details of seven adult myocarditis/inflammatory cardiomyopathy cases

	Patient - 1	Patient - 2	Patient - 3	Patient - 4	Patient - 5	Patient - 6	Patient - 7
**Age (years)**	61	48	68	76	48	61	27
**Sex**	Female	Male	Female	Male	Male	Male	Male
**Self-reported ethnicity**	Caucasian	Caucasian	Caucasian	Caucasian	Caucasian	Caucasian	Caucasian
**Maximum CRP (mg/L) [normal range < 3 mg/L]**	136	4	14.3	270.5	3	31.4	56.6
**Maximum cardiac Troponin (ng/L) [normal range:<14ng/L]**	353	48	610	34	20	318	2179
**Maximum NT-proBNP (ng/L) [normal range < 50 ng/L]**	38744	256	824	9004	8131	208886	266
**Ejection fraction on echocardiogram [normal range 50-70%]**	19%	40%	59%	20%	15%	33%	56%
**Significant Cardiac Magnetic Resonance Imaging findings**	Not done	late gadolinium enhancement	No significant abnormality	late gadolinium enhancement, perimyocarditis	late gadolinium enhancement	No significant abnormality	late gadolinium enhancement, edema, myocarditis
**Cardiac biopsy diagnosis**	inflammatory cardiomyopathy	inflammatory cardiomyopathy	acute myocarditis	inflammatory cardiomyopathy	inflammatory cardiomyopathy	inflammatory cardiomyopathy	inflammatory cardiomyopathy
**Final diagnosis**	terminal inflammatory cardiomyopathy	inflammatory cardiomyopathy	Immune Checkpoint Inhibitors-induced myocarditis	inflammatory cardiomyopathy	inflammatory cardiomyopathy	inflammatory cardiomyopathy	recurrent myocarditis

© 2023 Patel H et al. *JAMA Network Open.*

eFigure 1. IgG Immunohistochemistry images of adult myocarditis (positive control) repeated on 3 separate experiments

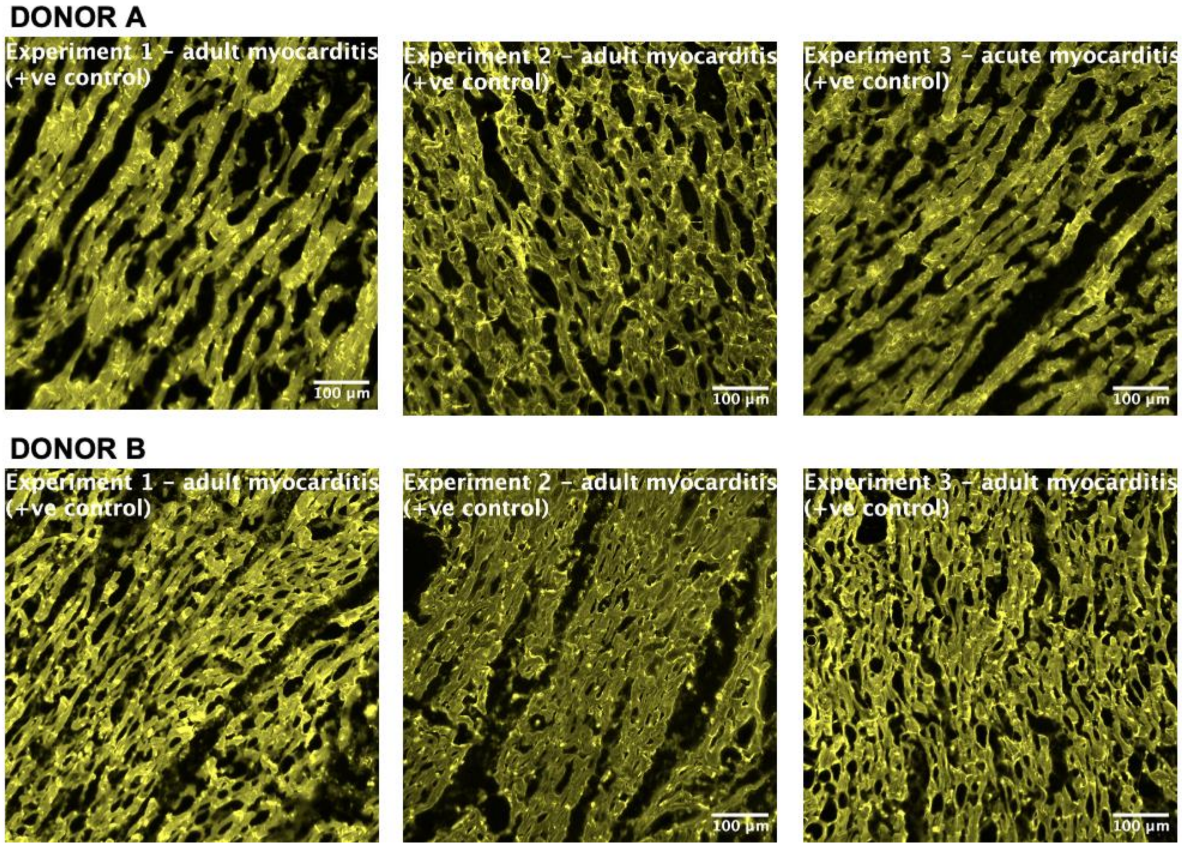

Supplementary eFigure 1: Immunohistochemistry images of cardiac tissue (Donor A and Donor B) treated with serum (1:50) from an adult with non-SARS-CoV-2 myocarditis and stained with Fluorescein isothiocyanate (FIT-C) conjugated anti-human IgG, repeated on three separate experiments. Images were taken on widefield microscope at using a 20x 0.8na objective.

© 2023 Patel H et al. *JAMA Network Open.*

eFigure 2. IgM immunohistochemistry images – Donor A

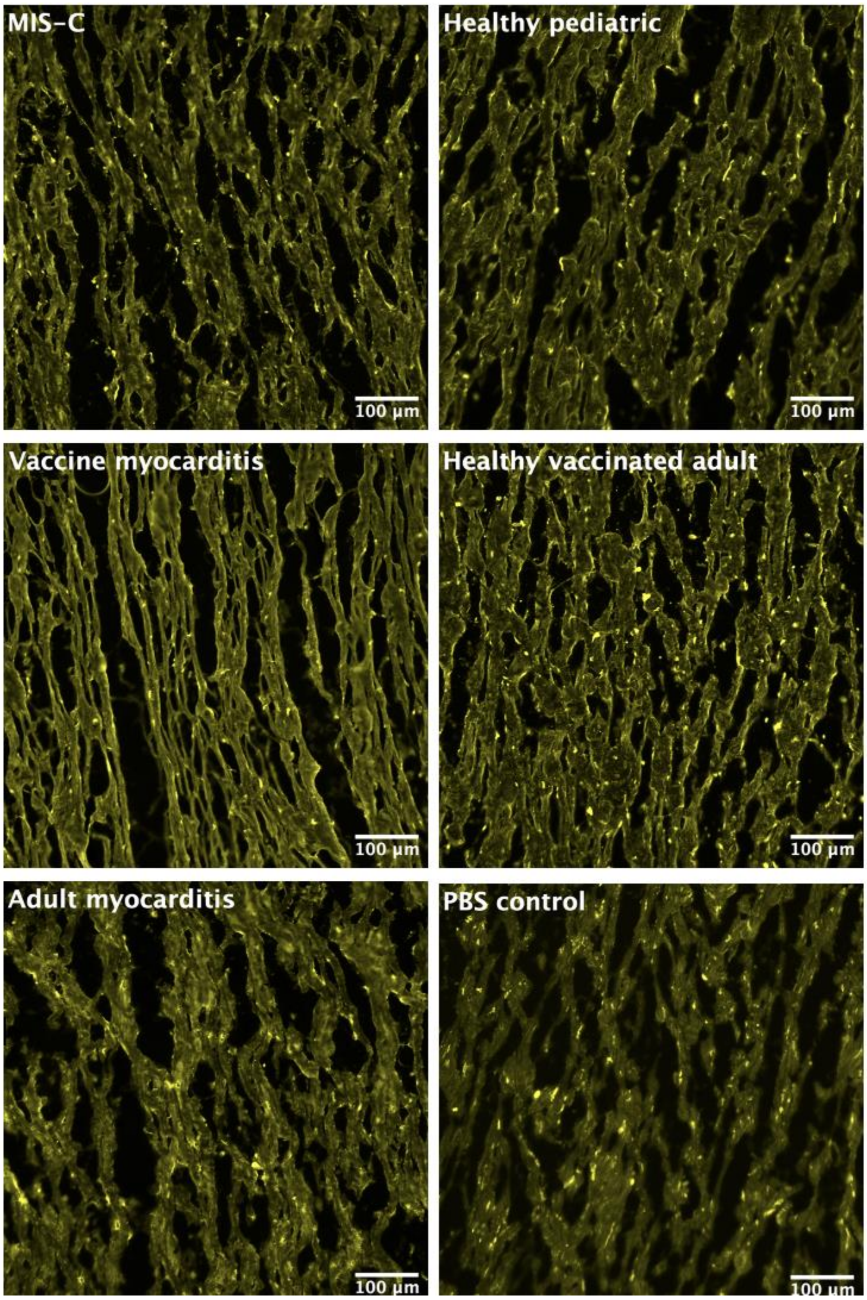

Supplementary eFigure 2: Immunohistochemistry images of cardiac tissue (Donor A) treated with serum (1:50) from patients and controls and stained with Fluorescein isothiocyanate (FIT-C) conjugated anti-human IgM. Images were taken on widefield microscope at using a 20x 0.8na objective. An example image from each of the following groups has been shown: Phosphate buffered saline (PBS) – negative control, healthy pre-pandemic paediatric control, healthy adults ≥ 14 days following COVID-19 vaccination, COVID-19 vaccine associated myocarditis and Multisystem Inflammatory Syndrome in Children (MIS-C).

© 2023 Patel H et al. *JAMA Network Open.*

eFigure 3. IgM immunohistochemistry images – Donor B

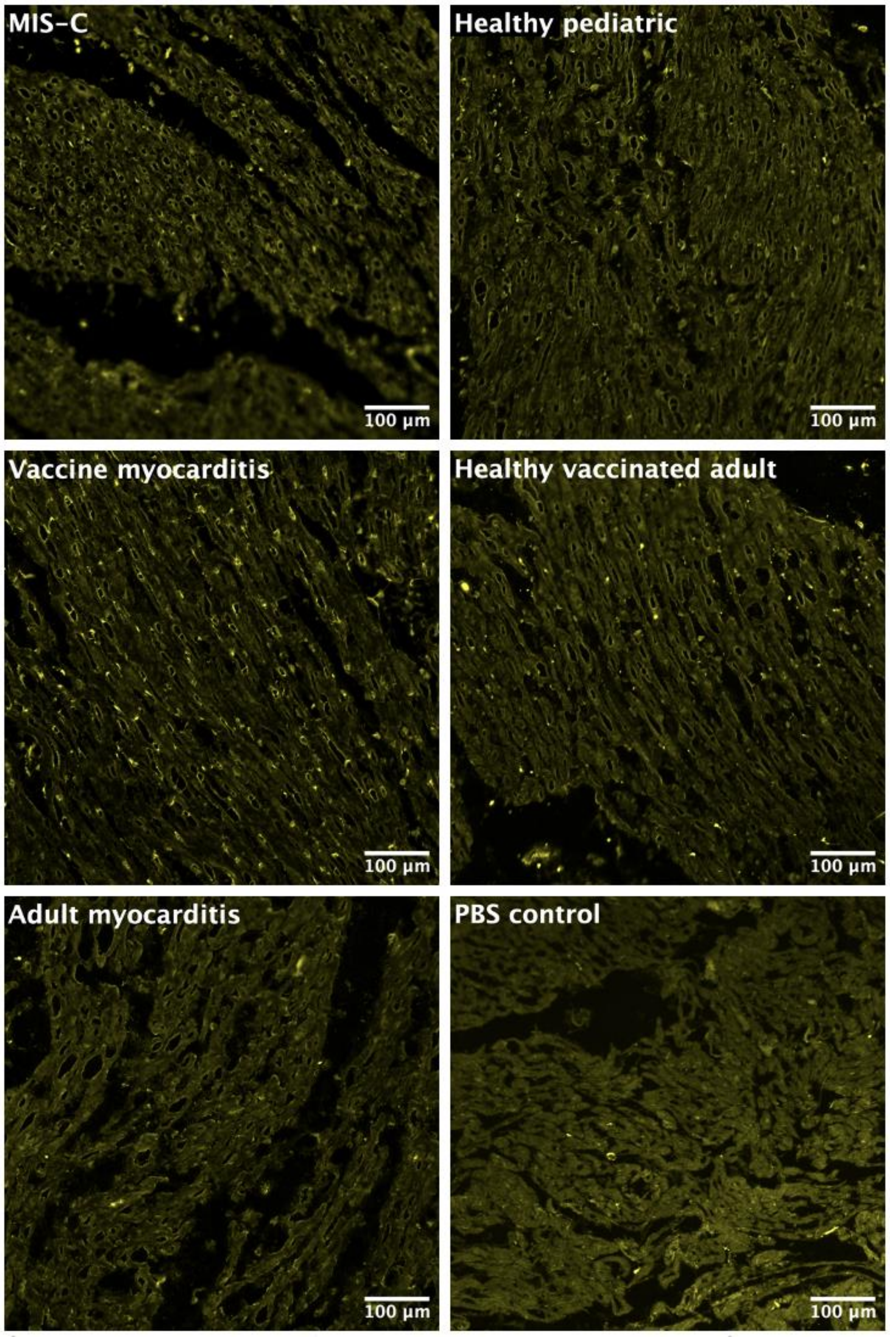

Supplementary eFigure 3: Immunohistochemistry images of cardiac tissue (Donor B) treated with serum (1:50) from patients and controls and stained with Fluorescein isothiocyanate (FIT-C) conjugated anti-human IgM. Images were taken on widefield microscope at using a 20x 0.8na objective. An example image from each of the following groups has been shown: Phosphate buffered saline (PBS) – negative control, healthy pre-pandemic paediatric control, healthy adults ≥ 14 days following COVID-19 vaccination, COVID-19 vaccine associated myocarditis and Multisystem Inflammatory Syndrome in Children (MIS-C).

© 2023 Patel H et al. *JAMA Network Open.*

eFigure 4. IgA immunohistochemistry images – Donor A

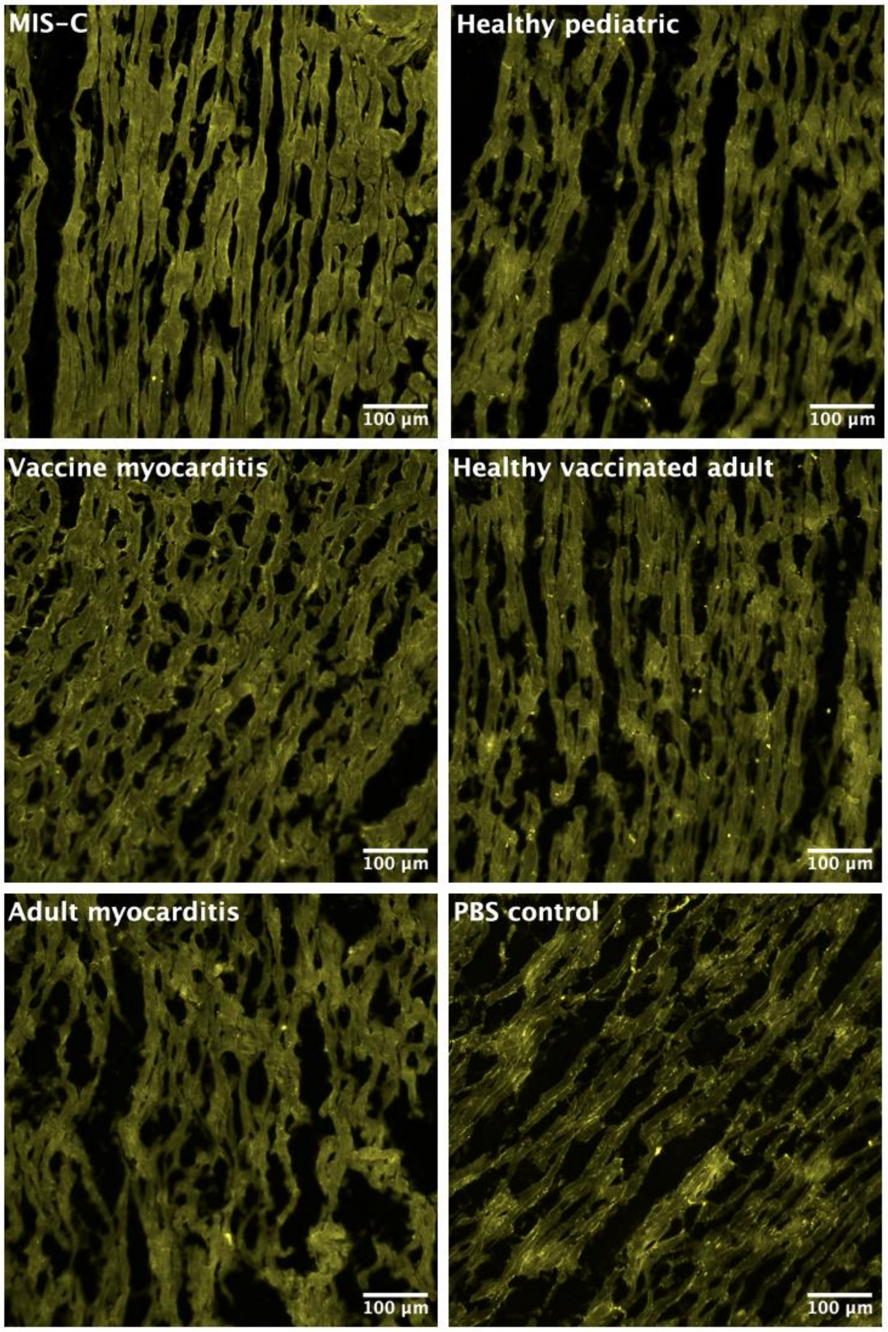

Supplementary eFigure 4: Immunohistochemistry images of cardiac tissue (Donor A) treated with serum (1:50) from patients and controls and stained with Fluorescein isothiocyanate (FIT-C) conjugated anti-human IgA. Images were taken on widefield microscope at using a 20x 0.8na objective. An example image from each of the following groups has been shown: Phosphate buffered saline (PBS) – negative control, healthy pre-pandemic paediatric control, healthy adults ≥ 14 days following COVID-19 vaccination, COVID-19 vaccine associated myocarditis and Multisystem Inflammatory Syndrome in Children (MIS-C).

© 2023 Patel H et al. *JAMA Network Open.*

eFigure 5. IgA immunohistochemistry images – Donor B

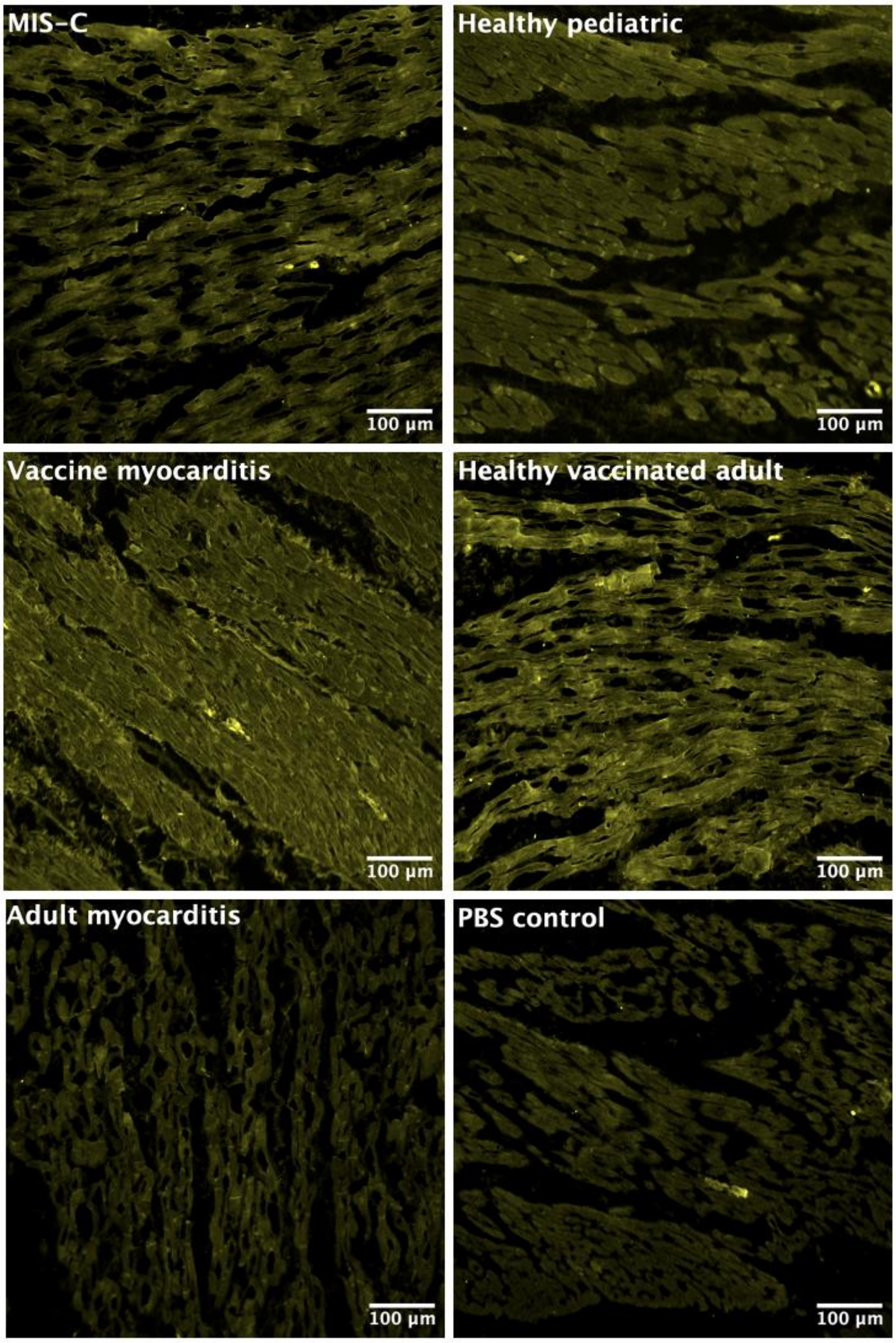

Supplementary eFigure 5: Immunohistochemistry images of cardiac tissue (Donor B) treated with serum (1:50) from patients and controls and stained with Fluorescein isothiocyanate (FIT-C) conjugated anti-human IgA. Images were taken on widefield microscope at using a 20x 0.8na objective. An example image from each of the following groups has been shown: Phosphate buffered saline (PBS) – negative control, healthy pre-pandemic paediatric control, healthy adults ≥ 14 days following COVID-19 vaccination, COVID-19 vaccine associated myocarditis and Multisystem Inflammatory Syndrome in Children (MIS-C).

© 2023 Patel H et al. *JAMA Network Open.*

eFigure 6. IgG immunohistochemistry images for adult myocarditis/inflammatory cardiomyopathy patients – Donor A

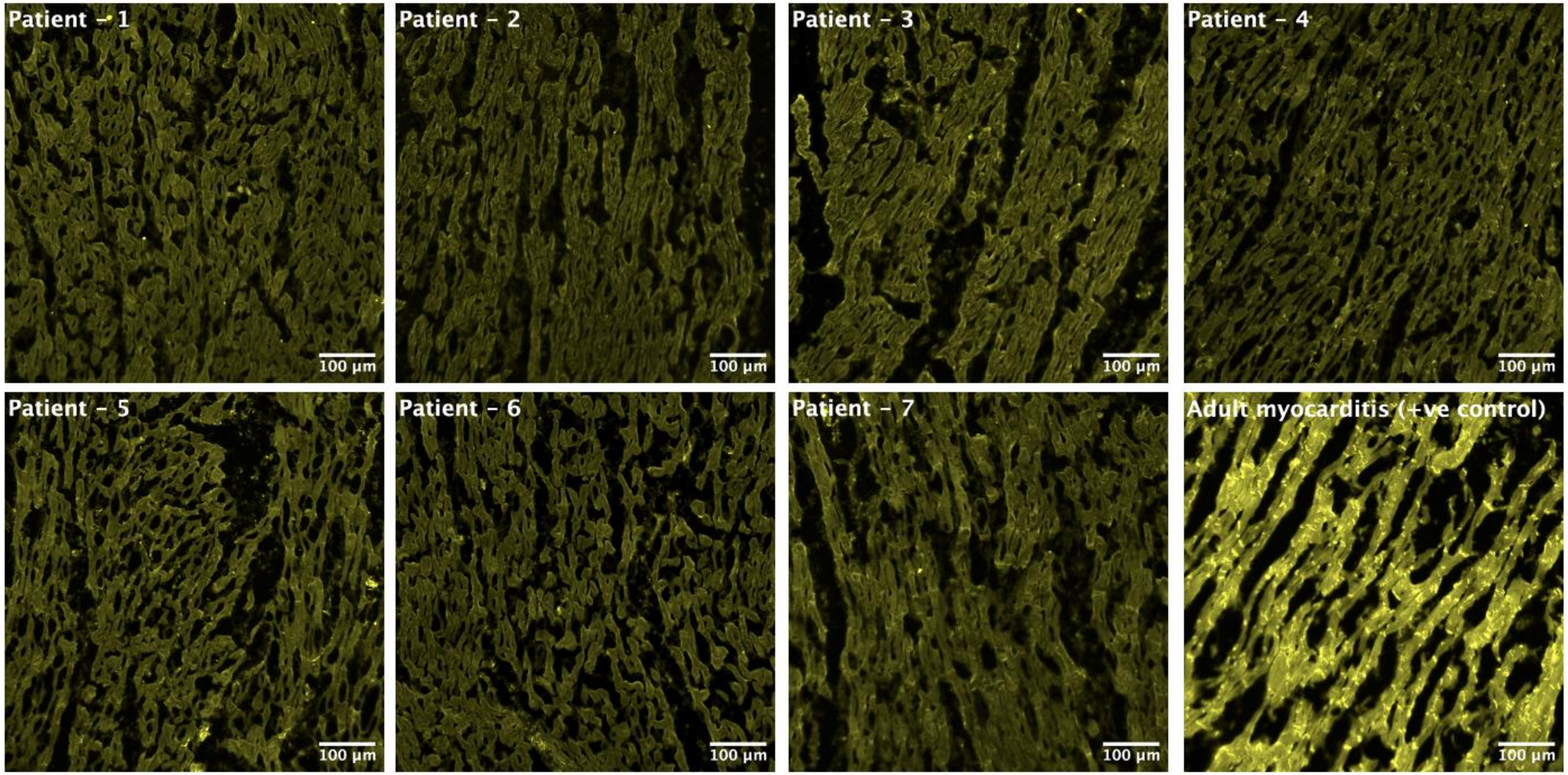

Supplementary eFigure 6: Immunohistochemistry images of cardiac tissue (Donor B) treated with serum (1:50) from adults with myocarditis/inflammatory cardiomyopathy stained with Fluorescein isothiocyanate (FIT-C) conjugated anti-human IgA. Images were taken on widefield microscope at using a 20x 0.8na objective. An example image from each patient has been shown.

© 2023 Patel H et al. *JAMA Network Open.*
