# Supplementary material for: Evaluation of Autoantibody Binding to Cardiac Tissue in Multisystem Inflammatory Syndrome in Children and COVID-19 Vaccination–Induced Myocarditis
Source: JAMA Netw Open. 2023 May 18;6(5):e2314291. doi: 10.1001/jamanetworkopen.2023.14291 (PMC10196878; doi:10.1001/jamanetworkopen.2023.14291)
Supplement: Supplement 2. — Nonauthor Collaborators [file jamanetwopen-e2314291-s002.pdf]

Supplemental Online Content: Nonauthor Collaborators
*First name, last name, and suffix (if applicable) are required and will appear in PubMed.

*Group Name(s): DIAMONDS consortium							
***First Name and Middle Initial(s)**	***Last Name**	***Suffix (eg, Jr, III)**	Academic Degrees	Institution	Location (city, state/province, country)	Role or Contribution, eg, chair, principal investigator	Group (if more than 1 Group listed in the byline) and/or Subgroup (eg, Steering Committee)
Aubrey	Cunnington			Imperial College London		Consortium member	
Jethro	Herberg			Imperial College London		Consortium member	
Myrsini	Kaforou			Imperial College London		Consortium member	
Victoria J.	Wright			Imperial College London		Consortium member	
Evangelos	Bellos			Imperial College London		Consortium member	
Claire	Broderick			Imperial College London		Consortium member	
Samuel	Channon-Wells			Imperial College London		Consortium member	
Samantha	Cooray			Imperial College London		Consortium member	
Tisham	De			Imperial College London		Consortium member	
Giselle	D’Souza			Imperial College London		Consortium member	
Leire	Estramiana Elorrieta			Imperial College London		Consortium member	
Diego	Estrada-Rivadeneyra			Imperial College London		Consortium member	
Rachel	Galassini			Imperial College London		Consortium member	
Dominic	Habgood-Coote			Imperial College London		Consortium member	
Shea	Hamilton			Imperial College London		Consortium member	
Heather	Jackson			Imperial College London		Consortium member	
James	Kavanagh			Imperial College London		Consortium member	
Ilana	Keren			Imperial College London		Consortium member	
Mahdi	Moradi Marjaneh			Imperial College London		Consortium member	
Stephanie	Menikou			Imperial College London		Consortium member	
Samuel	Nichols			Imperial College London		Consortium member	
Ruud	Nijman			Imperial College London		Consortium member	
Ivana	Pennisi			Imperial College London		Consortium member	
Oliver	Powell			Imperial College London		Consortium member	
Ruth	Reid			Imperial College London		Consortium member	
Priyen	Shah			Imperial College London		Consortium member	
Ortensia	Vito			Imperial College London		Consortium member	
Elizabeth	Whittaker			Imperial College London		Consortium member	
Clare	Wilson			Imperial College London		Consortium member	
Rebecca	Womersley			Imperial College London		Consortium member	
Amina	Abdulla			Imperial College Healthcare NHS Trust		Consortium member	
Sarah	Darnell			Imperial College Healthcare NHS Trust		Consortium member	

*First name, last name, and suffix (if applicable) are required and will appear in PubMed.

***First Name and Middle Initial(s)**	***Last Name**	***Suffix (eg, Jr, III)**	Academic Degrees	Institution	Location (city, state/province, country)	Role or Contribution, eg, chair, principal investigator	Group (if more than 1 Group listed in the byline) and/or Subgroup (eg, Steering Committee)
Sobia	Mustafa			Imperial College Healthcare NHS Trust		Consortium member	
Pantelis	Georgiou			Imperial College London		Consortium member	
Jesus-Rodriguez	Manzano			Imperial College London		Consortium member	
Nicolas	Moser			Imperial College London		Consortium member	
Michael	Carter			Evelina London Children's Hospital, Guy's and St Thomas' NHS Foundation Trust; King's College London		Consortium member	
Shane	Tibby			Evelina London Children's Hospital, Guy's and St Thomas' NHS Foundation Trust; King's College London		Consortium member	
Jonathan	Cohen			Evelina London Children's Hospital, Guy's and St Thomas' NHS Foundation Trust; King's College London		Consortium member	
Francesca	Davis			Evelina London Children's Hospital, Guy's and St Thomas' NHS Foundation Trust; King's College London		Consortium member	
Julia	Kenny			Evelina London Children's Hospital, Guy's and St Thomas' NHS Foundation Trust; King's College London		Consortium member	
Paul	Wellman			Evelina London Children's Hospital, Guy's and St Thomas' NHS Foundation Trust; King's College London		Consortium member	
Marie	White			Evelina London Children's Hospital, Guy's and St Thomas' NHS Foundation Trust; King's College London		Consortium member	

***First Name and Middle Initial(s)**	***Last Name**	***Suffix (eg, Jr, III)**	Academic Degrees	Institution	Location (city, state/province, country)	Role or Contribution, eg, chair, principal investigator	Group (if more than 1 Group listed in the byline) and/or Subgroup (eg, Steering Committee)
Matthew	Fish			Evelina London Children’s Hospital, Guy’s and St Thomas’ NHS Foundation Trust; King’s College London		Consortium member	
Aislinn	Jennings			Evelina London Children’s Hospital, Guy’s and St Thomas’ NHS Foundation Trust; King’s College London		Consortium member	
Manu	Shankar-Hari			Evelina London Children’s Hospital, Guy’s and St Thomas’ NHS Foundation Trust; King’s College London		Consortium member	
Katy	Fidler			University Hospitals Sussex		Consortium member	
Dan	Agranoff			University Hospitals Sussex		Consortium member	
Vivien	Richmond			University Hospitals Sussex		Consortium member	
Mathhew	Seal			University Hospitals Sussex		Consortium member	
Saul	Faust			University Hospital Southampton NHS		Consortium member	
Dan	Owen			University Hospital Southampton NHS Foundation Trust		Consortium member	
Ruth	Ensom			University Hospital Southampton NHS Foundation Trust		Consortium member	
Sarah	McKay			University Hospital Southampton NHS Foundation Trust		Consortium member	
Mariya	Shaji			University Hospital Southampton NHS Foundation Trust		Consortium member	
Rachel	Schranz			University Hospital Southampton NHS Foundation Trust		Consortium member	
Prita	Rughnani			Barts Health NHS Trust		Consortium member	
Amutha	Anpananthar			Barts Health NHS Trust		Consortium member	
Susan	Liebeschuetz			Barts Health NHS Trust		Consortium member	
Anna	Riddell			Barts Health NHS Trust		Consortium member	
Divya	Divakaran			Barts Health NHS Trust		Consortium member	
Louise	Han			Barts Health NHS Trust		Consortium member	
Nosheen	Khalid			Barts Health NHS Trust		Consortium member	
Ivone Lancoma	Malcolm			Barts Health NHS Trust		Consortium member	

*First name, last name, and suffix (if applicable) are required and will appear in PubMed.

***First Name and Middle Initial(s)**	***Last Name**	***Suffix (eg, Jr, III)**	Academic Degrees	Institution	Location (city, state/province, country)	Role or Contribution, eg, chair, principal investigator	Group (if more than 1 Group listed in the byline) and/or Subgroup (eg, Steering Committee)
Jessica	Schofield			Barts Health NHS Trust		Consortium member	
Teresa	Simagan			Barts Health NHS Trust		Consortium member	
Mark	Peters			Great Ormond Street Hospital for Children NHS Foundation Trust		Consortium member	
Alasdair	Bamford			Great Ormond Street Hospital for Children NHS Foundation Trust		Consortium member	
Lauran	O’Neill			Great Ormond Street Hospital for Children NHS Foundation Trust		Consortium member	
Nazima	Pathan			Cambridge University Hospitals NHS Foundation Trust		Consortium member	
Esther	Daubney			Cambridge University Hospitals NHS Foundation Trust		Consortium member	
Deborah	White			Cambridge University Hospitals NHS Foundation Trust		Consortium member	
Melissa	Heightman			University College London Hospitals NHS Foundation Trust		Consortium member	
Sarah	Eisen			University College London Hospitals NHS Foundation Trust		Consortium member	
Terry	Segal			University College London Hospitals NHS Foundation Trust		Consortium member	
Lucy	Wellings			University College London Hospitals NHS Foundation Trust		Consortium member	
Simon	B Drysdale			St George’s University Hospitals NHS Foundation Trust		Consortium member	
Nicole	Branch			St George’s University Hospitals NHS Foundation Trust		Consortium member	
Lisa	Hamzah			St George’s University Hospitals NHS Foundation Trust		Consortium member	
Heather	Jarman			St George’s University Hospitals NHS Foundation Trust		Consortium member	
Maggie	Nyirenda			Lewisham and Greenwich NHS Trust		Consortium member	
Lisa	Capozzi			Lewisham and Greenwich NHS Trust		Consortium member	
Emma	Gardiner			Lewisham and Greenwich NHS Trust		Consortium member	
Robert	Moots			Liverpool University Hospitals NHS Foundation Trust		Consortium member	

Supplemental Online Content: Nonauthor Collaborators
*First name, last name, and suffix (if applicable) are required and will appear in PubMed.

*First Name and Middle Initial(s)	*Last Name	*Suffix (eg, Jr, III)	Academic Degrees	Institution	Location (city, state/province, country)	Role or Contribution, eg, chair, principal investigator	Group (if more than 1 Group listed in the byline) and/or Subgroup (eg, Steering Committee)
Magda	Nasher			Liverpool University Hospitals NHS Foundation Trust		Consortium member	
Anita	Hanson			Liverpool University Hospitals NHS Foundation Trust		Consortium member	
Michelle	Linforth			Liverpool University Hospitals NHS Foundation Trust		Consortium member	
Sean	O'Riordan			Leeds Teaching Hospitals NHS Trust		Consortium member	
Donna	Ellis			Leeds Teaching Hospitals NHS Trust		Consortium member	
Akash	Deep			King's College Hospital NHS Foundation Trust		Consortium member	
Ivan	Caro			King's College Hospital NHS Foundation Trust		Consortium member	
Fiona	Shackley			Sheffield Children's NHS Foundation Trust		Consortium member	
Arianna	Bellini			Sheffield Children's NHS Foundation Trust		Consortium member	
Stuart	Gormley			Sheffield Children's NHS Foundation Trust		Consortium member	
Samira	Neshat			University Hospitals of Leicester NHS Foundation Trust		Consortium member	
Barnaby	Scholefield			Birmingham Women's and Children's Hospital NHS Foundation Trust		Consortium member	
Ceri	Robbins			Birmingham Women's and Children's Hospital NHS Foundation Trust		Consortium member	
Helen	Winmill			Birmingham Women's and Children's Hospital NHS Foundation Trust		Consortium member	
Stéphane C	Paulus			University of Oxford Partner Children's Hospital, John Radcliffe Hospital		Consortium member	
Andrew J	Pollard			University of Oxford Partner Children's		Consortium member	
Mark	Anthony			University of Oxford Partner Children's Hospital, John Radcliffe Hospital		Consortium member	
Sarah	Hopton			University of Oxford Partner Children's Hospital, John Radcliffe Hospital		Consortium member	

Supplemental Online Content: Nonauthor Collaborators
*First name, last name, and suffix (if applicable) are required and will appear in PubMed.

*First Name and Middle Initial(s)	*Last Name	*Suffix (eg, Jr, III)	Academic Degrees	Institution	Location (city, state/province, country)	Role or Contribution, eg, chair, principal investigator	Group (if more than 1 Group listed in the byline) and/or Subgroup (eg, Steering Committee)
Danielle	Miller			University of Oxford Partner Children's Hospital, John Radcliffe Hospital		Consortium member	
Zoe	Oliver			University of Oxford Partner Children's Hospital, John Radcliffe Hospital		Consortium member	
Sally	Beer			University of Oxford Partner Children's Hospital, John Radcliffe Hospital		Consortium member	
Bryony	Ward			University of Oxford Partner Children's Hospital, John Radcliffe Hospital		Consortium member	
Shrijana	Shrestha			University of Oxford, Nepal Site		Consortium member	
Meeru	Gurung			University of Oxford, Nepal Site		Consortium member	
Puja	Amatya			University of Oxford, Nepal Site		Consortium member	
Bhishma	Pokhrel			University of Oxford, Nepal Site		Consortium member	
Sanjeev Man	Bijukchhe			University of Oxford, Nepal Site		Consortium member	
Madhav Chandra	Gautam			University of Oxford, Nepal Site		Consortium member	
Sarah	Kelly			University of Oxford, Nepal Site		Consortium member	
Peter	O'Reilly			University of Oxford, Nepal Site		Consortium member	
Sonu	Shrestha			University of Oxford, Nepal Site		Consortium member	
Federico	Martinón-Torres			Hospital Clínico Universitario de Santiago, Santiago de Compostela		Consortium member	
Antonio	Salas			Hospital Clínico Universitario de Santiago, Santiago de Compostela		Consortium member	
Fernando Álvez	González			Hospital Clínico Universitario de Santiago, Santiago de Compostela		Consortium member	
Sonia Ares	Gómez			Hospital Clínico Universitario de Santiago, Santiago de Compostela		Consortium member	
Xabier	Bello			Hospital Clínico Universitario de Santiago, Santiago de Compostela		Consortium member	
Mirian Ben	García			Hospital Clínico Universitario de Santiago, Santiago de Compostela		Consortium member	
Fernando Caamaño	Viña			Hospital Clínico Universitario de Santiago, Santiago de Compostela		Consortium member	

*First Name and Middle Initial(s)	*Last Name	*Suffix (eg, Jr, III)	Academic Degrees	Institution	Location (city, state/province, country)	Role or Contribution, eg, chair, principal investigator	Group (if more than 1 Group listed in the byline) and/or Subgroup (eg, Steering Committee)
Sandra	Carnota			Hospital Clínico Universitario de Santiago, Santiago de Compostela		Consortium member	
María José	Curras-Tuala			Hospital Clínico Universitario de Santiago, Santiago de Compostela		Consortium member	
Ana Dacosta	Urbieta			Hospital Clínico Universitario de Santiago, Santiago de Compostela		Consortium member	
Carlos Durán	Suárez			Hospital Clínico Universitario de Santiago, Santiago de Compostela		Consortium member	
Isabel Ferreiros	Vidal			Hospital Clínico Universitario de Santiago, Santiago de Compostela		Consortium member	
Luisa García	Vicente			Hospital Clínico Universitario de Santiago, Santiago de Compostela		Consortium member	
Alberto	Gómez-Carballa			Hospital Clínico Universitario de Santiago, Santiago de Compostela		Consortium member	
Jose Gómez	Rial			Hospital Clínico Universitario de Santiago, Santiago de Compostela		Consortium member	
Pilar Leboráns	Iglesias			Hospital Clínico Universitario de Santiago, Santiago de Compostela		Consortium member	
Narmeen	Mallah			Hospital Clínico Universitario de Santiago, Santiago de Compostela		Consortium member	
Nazareth	Martinón-Torres			Hospital Clínico Universitario de Santiago, Santiago de Compostela		Consortium member	
José María	Martinón			Hospital Clínico Universitario de Santiago, Santiago de Compostela		Consortium member	
Belén Mosquera Pérez	Sánchez			Hospital Clínico Universitario de Santiago, Santiago de Compostela		Consortium member	
Jacobo	Pardo-Seco			Hospital Clínico Universitario de Santiago, Santiago de Compostela		Consortium member	
Sara	Pischedda			Hospital Clínico Universitario de Santiago, Santiago de Compostela		Consortium member	
Sara Rey	Vázquez			Hospital Clínico Universitario de Santiago, Santiago de Compostela		Consortium member	
Irene Rivero	Calle			Hospital Clínico Universitario de Santiago, Santiago de Compostela		Consortium member	
Carmen	Rodríguez-Tenreiro			Hospital Clínico Universitario de Santiago, Santiago de Compostela		Consortium member	

Supplemental Online Content: Nonauthor Collaborators
*First name, last name, and suffix (if applicable) are required and will appear in PubMed.

***First Name and Middle Initial(s)**	***Last Name**	***Suffix (eg, Jr, III)**	Academic Degrees	Institution	Location (city, state/province, country)	Role or Contribution, eg, chair, principal investigator	Group (if more than 1 Group listed in the byline) and/or Subgroup (eg, Steering Committee)
Lorenzo	Redondo-Collazo			Hospital Clínico Universitario de Santiago, Santiago de Compostela		Consortium member	
Antonio	Salas			Hospital Clínico Universitario de Santiago, Santiago de Compostela		Consortium member	
Sonia Serén	Fernández			Hospital Clínico Universitario de Santiago, Santiago de Compostela		Consortium member	
Marisol Vilas	Iglesias			Hospital Clínico Universitario de Santiago, Santiago de Compostela		Consortium member	
Enitan D	Carrol			University of Liverpool Institute of Infection, Veterinary and Ecological Sciences; Alder Hey Children's Hospital, Liverpool		Consortium member	
Elizabeth	Cocklin			University of Liverpool Institute of Infection, Veterinary and Ecological Sciences; Alder Hey Children's Hospital, Liverpool		Consortium member	
Rebecca	Beckley			University of Liverpool Institute of Infection, Veterinary and Ecological Sciences; Alder Hey Children's Hospital, Liverpool		Consortium member	
Abbey	Bracken			University of Liverpool Institute of Infection, Veterinary and Ecological Sciences; Alder Hey Children's Hospital, Liverpool		Consortium member	
Ceri	Evans			University of Liverpool Institute of Infection, Veterinary and Ecological Sciences; Alder Hey Children's Hospital, Liverpool		Consortium member	
Aakash	Khanijau			University of Liverpool Institute of Infection, Veterinary and Ecological Sciences; Alder Hey Children's Hospital, Liverpool		Consortium member	
Rebecca	Lenihan			University of Liverpool Institute of Infection, Veterinary and Ecological Sciences; Alder Hey Children's Hospital, Liverpool		Consortium member	

*First name, last name, and suffix (if applicable) are required and will appear in PubMed.

***First Name and Middle Initial(s)**	***Last Name**	***Suffix (eg, Jr, III)**	Academic Degrees	Institution	Location (city, state/province, country)	Role or Contribution, eg, chair, principal investigator	Group (if more than 1 Group listed in the byline) and/or Subgroup (eg, Steering Committee)
Nadia	Lewis-Burke			University of Liverpool Institute of Infection, Veterinary and Ecological Sciences; Alder Hey Children's Hospital, Liverpool		Consortium member	
Karen	Newall			Alder Hey Children's Hospital, Liverpool		Consortium member	
Sam	Romaine			Alder Hey Children's Hospital, Liverpool		Consortium member	
Jennifer	Whitbread			Alder Hey Children's Hospital, Liverpool		Consortium member	
Maria	Tsolia			National and Kapodistrian University of Athens		Consortium member	
Irini	Eleftheriou			National and Kapodistrian University of Athens		Consortium member	
Nikos	Spyridis			National and Kapodistrian University of Athens		Consortium member	
Maria	Tambouratzi			National and Kapodistrian University of Athens		Consortium member	
Despoina	Maritsi			National and Kapodistrian University of Athens		Consortium member	
Antonios	Marmarinos			National and Kapodistrian University of Athens		Consortium member	
Marietta	Xagorari			National and Kapodistrian University of Athens		Consortium member	
Lourida	Panagiota			National and Kapodistrian University of Athens		Consortium member	
Pefanis	Aggelos			National and Kapodistrian University of Athens		Consortium member	
Akinosoglou	Karolina			National and Kapodistrian University of Athens		Consortium member	
Gogos	Charalambos			National and Kapodistrian University of Athens		Consortium member	
Maragos	Markos			National and Kapodistrian University of Athens		Consortium member	
Voulgarelis	Michalis			National and Kapodistrian University of Athens		Consortium member	

Supplemental Online Content: Nonauthor Collaborators
*First name, last name, and suffix (if applicable) are required and will appear in PubMed.

***First Name and Middle Initial(s)**	***Last Name**	***Suffix (eg, Jr, III)**	Academic Degrees	Institution	Location (city, state/province, country)	Role or Contribution, eg, chair, principal investigator	Group (if more than 1 Group listed in the byline) and/or Subgroup (eg, Steering Committee)
Stergiou	Ioanna			National and Kapodistrian University of Athens		Consortium member	
Marieke	Emonts			Newcastle upon Tyne Hospitals NHS Foundation Trust and Newcastle University (UK)		Consortium member	
Emma	Lim			Newcastle upon Tyne Hospitals NHS Foundation Trust and Newcastle University (UK)		Consortium member	
John	Isaacs			Newcastle upon Tyne Hospitals NHS Foundation Trust and Newcastle University (UK)		Consortium member	
Kathryn	Bell			Newcastle upon Tyne Hospitals NHS Foundation Trust and Newcastle University (UK)		Consortium member	
Stephen	Crulley			Newcastle upon Tyne Hospitals NHS Foundation Trust and Newcastle University (UK)		Consortium member	
Daniel	Fabian			Newcastle upon Tyne Hospitals NHS Foundation Trust and Newcastle University (UK)		Consortium member	
Evelyn	Thomson			Newcastle upon Tyne Hospitals NHS Foundation Trust and Newcastle University (UK)		Consortium member	
Diane	Wallia			Newcastle upon Tyne Hospitals NHS Foundation Trust and Newcastle University (UK)		Consortium member	
Caroline	Miller			Newcastle upon Tyne Hospitals NHS Foundation Trust and Newcastle University (UK)		Consortium member	
Ashley	Bell			Newcastle upon Tyne Hospitals NHS Foundation Trust and Newcastle University (UK)		Consortium member	
Fabian J S	van der Velden			Newcastle upon Tyne Hospitals NHS Foundation Trust and Newcastle University (UK)		Consortium member	

Supplemental Online Content: Nonauthor Collaborators
*First name, last name, and suffix (if applicable) are required and will appear in PubMed.

***First Name and Middle Initial(s)**	***Last Name**	***Suffix (eg, Jr, III)**	Academic Degrees	Institution	Location (city, state/province, country)	Role or Contribution, eg, chair, principal investigator	Group (if more than 1 Group listed in the byline) and/or Subgroup (eg, Steering Committee)
Geoff	Shenton			Newcastle upon Tyne Hospitals NHS Foundation Trust and Newcastle University (UK)		Consortium member	
Ashley	Price			Newcastle upon Tyne Hospitals NHS Foundation Trust and Newcastle University (UK)		Consortium member	
Owen	Treloar			Newcastle upon Tyne Hospitals NHS Foundation Trust and Newcastle University (UK)		Consortium member	
Daisy	Thomas			Newcastle upon Tyne Hospitals NHS Foundation Trust and Newcastle University (UK)		Consortium member	
Pablo	Rojo			Servicio Madrileño de Salud (SERMAS) - Fundación Biomédica del Hospital Universitario 12 de Octubre (FIB-H12O)		Consortium member	
Cristina	Epalza			Servicio Madrileño de Salud (SERMAS) - Fundación Biomédica del Hospital Universitario 12 de Octubre (FIB-H12O)		Consortium member	
Serena	Villaverde			Servicio Madrileño de Salud (SERMAS) - Fundación Biomédica del Hospital Universitario 12 de Octubre (FIB-H12O)		Consortium member	
Sonia	Márquez			Servicio Madrileño de Salud (SERMAS) - Fundación Biomédica del Hospital Universitario 12 de Octubre (FIB-H12O)		Consortium member	
Manuel	Gijón			Servicio Madrileño de Salud (SERMAS) - Fundación Biomédica del Hospital Universitario 12 de Octubre (FIB-H12O)		Consortium member	
Fátima	Machín			Servicio Madrileño de Salud (SERMAS) - Fundación Biomédica del Hospital Universitario 12 de Octubre (FIB-H12O)		Consortium member	

*First Name and Middle Initial(s)	*Last Name	*Suffix (eg, Jr, III)	Academic Degrees	Institution	Location (city, state/province, country)	Role or Contribution, eg, chair, principal investigator	Group (if more than 1 Group listed in the byline) and/or Subgroup (eg, Steering Committee)
Laura	Cabello			Servicio Madrileño de Salud (SERMAS) - Fundación Biomédica del Hospital Universitario 12 de Octubre (FIB-H12O)		Consortium member	
Irene	Hernández			Servicio Madrileño de Salud (SERMAS) - Fundación Biomédica del Hospital Universitario 12 de Octubre (FIB-H12O)		Consortium member	
Lourdes	Gutiérrez			Servicio Madrileño de Salud (SERMAS) - Fundación Biomédica del Hospital Universitario 12 de Octubre (FIB-H12O)		Consortium member	
Ángela	Manzanares			Servicio Madrileño de Salud (SERMAS) - Fundación Biomédica del Hospital Universitario 12 de Octubre (FIB-H12O)		Consortium member	
Taco W	Kuijpers			Amsterdam University Medical Center		Consortium member	
Martijn	van de Kuip			Amsterdam University Medical Center		Consortium member	
Marceline	van Furth			Amsterdam University Medical Center		Consortium member	
Merlijn	van den Berg			Amsterdam University Medical Center		Consortium member	
Giske	Biesbroek			Amsterdam University Medical Center		Consortium member	
Floris	Verkuil			Amsterdam University Medical Center		Consortium member	
Carlijn	van der Zee			Amsterdam University Medical Center		Consortium member	
Dasja	Pajkrt			Amsterdam University Medical Center		Consortium member	
Michael Boele	van Hensbroek			Amsterdam University Medical Center		Consortium member	
Dieneke	Schonenberg			Amsterdam University Medical Center		Consortium member	

*First name, last name, and suffix (if applicable) are required and will appear in PubMed.

***First Name and Middle Initial(s)**	***Last Name**	***Suffix (eg, Jr, III)**	Academic Degrees	Institution	Location (city, state/province, country)	Role or Contribution, eg, chair, principal investigator	Group (if more than 1 Group listed in the byline) and/or Subgroup (eg, Steering Committee)
Mariken	Gruppen			Amsterdam University Medical Center		Consortium member	
Sietse	Nagelkerke			Amsterdam University Medical Center		Consortium member	
MachielH	Jansen			Amsterdam University Medical Center		Consortium member	
Ines	Goetschalckx			Amsterdam University Medical Center		Consortium member	
Lorenza	Romani			Bambino Gesù Children’s Hospital, Rome		Consortium member	
Maia	De Luca			Bambino Gesù Children’s Hospital, Rome		Consortium member	
Sara	Chiurchiù			Bambino Gesù Children’s Hospital, Rome		Consortium member	
Costanza	Tripiciano			Bambino Gesù Children’s Hospital, Rome		Consortium member	
Stefania	Mercadante			Bambino Gesù Children’s Hospital, Rome		Consortium member	
Clementien L	Vermont			ERASMUS MC-Sophia Children’s Hospital		Consortium member	
Henriëtte A	Moll			ERASMUS MC-Sophia Children’s Hospital		Consortium member	
Dorine M	Borensztajn			ERASMUS MC-Sophia Children’s Hospital		Consortium member	
Nienke N	Hagedoorn			ERASMUS MC-Sophia Children’s Hospital		Consortium member	
Chantal	Tan			ERASMUS MC-Sophia Children’s Hospital		Consortium member	
Joany	Zachariasse			ERASMUS MC-Sophia Children’s Hospital		Consortium member	
W	Dik			ERASMUS MC-Sophia Children’s Hospital		Consortium member	
Ching-Fen	Shen			National Cheng Kung University Hospital, College of Medicine, National Cheng Kung University, Taiwan		Consortium member	

Supplemental Online Content: Nonauthor Collaborators
*First name, last name, and suffix (if applicable) are required and will appear in PubMed.

***First Name and Middle Initial(s)**	***Last Name**	***Suffix (eg, Jr, III)**	Academic Degrees	Institution	Location (city, state/province, country)	Role or Contribution, eg, chair, principal investigator	Group (if more than 1 Group listed in the byline) and/or Subgroup (eg, Steering Committee)
Dace	Zavadska			Riga Stradins University		Consortium member	
Sniedze	Laivacuma			Riga Stradins University		Consortium member	
Aleksandra	Rudzate			Riga Stradins University		Consortium member	
Diana	Stoldere			Riga Stradins University		Consortium member	
Arta	Barzdina			Riga Stradins University		Consortium member	
Elza	Barzdina			Riga Stradins University		Consortium member	
Sniedze	Laivacuma			Riga Stradins University		Consortium member	
Monta	Madelane			Riga Stradins University		Consortium member	
Dagne	Gravele			Children clinical university hospital, Riga		Consortium member	
Dace	Svilz			Children clinical university hospital, Riga		Consortium member	
Romain	Basmaci			Service de Pédiatrie-Urgences, AP-HP, Hôpital Louis-Mourier, F-92700 Colombes, France		Consortium member	
Noémie	Lachaume			Service de Pédiatrie-Urgences, AP-HP, Hôpital Louis-Mourier, F-92700 Colombes, France		Consortium member	
Pauline	Bories			Service de Pédiatrie-Urgences, AP-HP, Hôpital Louis-Mourier, F-92700 Colombes, France		Consortium member	
RajaBen	Tkhayat			Service de Pédiatrie-Urgences, AP-HP, Hôpital Louis-Mourier, F-92700 Colombes, France		Consortium member	
Laura	Chériaux			Service de Pédiatrie-Urgences, AP-HP, Hôpital Louis-Mourier, F-92700 Colombes, France		Consortium member	
Juraté	Davoust			Service de Pédiatrie-Urgences, AP-HP, Hôpital Louis-Mourier, F-92700 Colombes, France		Consortium member	
Kim-Thanh	Ong			Service de Pédiatrie-Urgences, AP-HP, Hôpital Louis-Mourier, F-92700 Colombes, France		Consortium member	
Marie	Cotillon			Service de Pédiatrie-Urgences, AP-HP, Hôpital Louis-Mourier, F-92700 Colombes, France		Consortium member	

Supplemental Online Content: Nonauthor Collaborators
*First name, last name, and suffix (if applicable) are required and will appear in PubMed.

***First Name and Middle Initial(s)**	***Last Name**	***Suffix (eg, Jr, III)**	Academic Degrees	Institution	Location (city, state/province, country)	Role or Contribution, eg, chair, principal investigator	Group (if more than 1 Group listed in the byline) and/or Subgroup (eg, Steering Committee)
Thibault	de Groc			Service de Pédiatrie-Urgences, AP-HP, Hôpital Louis-Mourier, F-92700 Colombes, France		Consortium member	
Sébastien	Le			Service de Pédiatrie-Urgences, AP-HP, Hôpital Louis-Mourier, F-92700 Colombes, France		Consortium member	
Nathalie	Vergnault			Service de Pédiatrie-Urgences, AP-HP, Hôpital Louis-Mourier, F-92700 Colombes, France		Consortium member	
Hélène	Sée			Service de Pédiatrie-Urgences, AP-HP, Hôpital Louis-Mourier, F-92700 Colombes, France		Consortium member	
Laure	Cohen			Service de Pédiatrie-Urgences, AP-HP, Hôpital Louis-Mourier, F-92700 Colombes, France		Consortium member	
Alice	de Tugny			Service de Pédiatrie-Urgences, AP-HP, Hôpital Louis-Mourier, F-92700 Colombes, France		Consortium member	
Nevena	Danekova			Service de Pédiatrie-Urgences, AP-HP, Hôpital Louis-Mourier, F-92700 Colombes, France		Consortium member	
Marine	Mommert-Tripon			BioMérieux - Open Innovation & Partnerships Department, Lyon, Franceérieux		Consortium member	
Karen	Brengel-Pesce			BioMérieux - Open Innovation & Partnerships Department, Lyon, Franceérieux		Consortium member	
Marko	Pokorn			University Medical Centre Ljubljana, Slovenia		Consortium member	
Mojca	Kolnik			University Medical Centre Ljubljana, Slovenia		Consortium member	
Tadej	Avčin			University Medical Centre Ljubljana, Slovenia		Consortium member	
Tanja	Avramoska			University Medical Centre Ljubljana, Slovenia		Consortium member	

Supplemental Online Content: Nonauthor Collaborators
*First name, last name, and suffix (if applicable) are required and will appear in PubMed.

***First Name and Middle Initial(s)**	***Last Name**	***Suffix (eg, Jr, III)**	Academic Degrees	Institution	Location (city, state/province, country)	Role or Contribution, eg, chair, principal investigator	Group (if more than 1 Group listed in the byline) and/or Subgroup (eg, Steering Committee)
Natalija	Bahovec			University Medical Centre Ljubljana, Slovenia		Consortium member	
Petra	Bogovič			University Medical Centre Ljubljana, Slovenia		Consortium member	
Lidija	Kitanovski			University Medical Centre Ljubljana, Slovenia		Consortium member	
Mirijam	Nahtigal			University Medical Centre Ljubljana, Slovenia		Consortium member	
Lea	Papst			University Medical Centre Ljubljana, Slovenia		Consortium member	
Tina	Plankar Srovin			University Medical Centre Ljubljana, Slovenia		Consortium member	
Franc	Strle			University Medical Centre Ljubljana, Slovenia		Consortium member	
Katarina	Vincek			University Medical Centre Ljubljana, Slovenia		Consortium member	
Michiel	van der Flier			University Medical Center Utrecht, Utrecht		Consortium member	
Wim J E	Tissing			University Medical Center Utrecht, Utrecht		Consortium member	
Roelie M	Wösten-van Asperen			University Medical Center Utrecht, Utrecht		Consortium member	
Sebastiaan J	Vastert			University Medical Center Utrecht, Utrecht		Consortium member	
Daniel C	Vijlbrief			University Medical Center Utrecht, Utrecht		Consortium member	
Louis J	Bont			University Medical Center Utrecht, Utrecht		Consortium member	
Coco R	Beudeker			University Medical Center Utrecht, Utrecht		Consortium member	
Philipp	Agyeman			Inselspital, Bern University Hospital, University of Bern, Switzerland		Consortium member	
Luregn	Schlapbach			Inselspital, Bern University Hospital, University of Bern, Switzerland		Consortium member	
Christoph	Aebi			Inselspital, Bern University Hospital, University of Bern, Switzerland		Consortium member	

Supplemental Online Content: Nonauthor Collaborators
*First name, last name, and suffix (if applicable) are required and will appear in PubMed.

***First Name and Middle Initial(s)**	***Last Name**	***Suffix (eg, Jr, III)**	Academic Degrees	Institution	Location (city, state/province, country)	Role or Contribution, eg, chair, principal investigator	Group (if more than 1 Group listed in the byline) and/or Subgroup (eg, Steering Committee)
Mariama	Usman			Inselspital, Bern University Hospital, University of Bern, Switzerland		Consortium member	
Stefanie	Schlüchter			Inselspital, Bern University Hospital, University of Bern, Switzerland		Consortium member	
Verena	Wyss			Inselspital, Bern University Hospital, University of Bern, Switzerland		Consortium member	
Nina	Schöbi			Inselspital, Bern University Hospital, University of Bern, Switzerland		Consortium member	
Elisa	Zimmermann			Inselspital, Bern University Hospital, University of Bern, Switzerland		Consortium member	
Marion	Meier			Inselspital, Bern University Hospital, University of Bern, Switzerland		Consortium member	
Kathrin	Weber			Inselspital, Bern University Hospital, University of Bern, Switzerland		Consortium member	
Colin	Fink			Micropathology Ltd, The Venture Center, University of Warwick Science Park, Sir William Lyons Road, Coventry		Consortium member	
Marie	Voice			Micropathology Ltd, The Venture Center, University of Warwick Science Park, Sir William Lyons Road, Coventry		Consortium member	
Leo	Calvo-Bado			Micropathology Ltd, The Venture Center, University of Warwick Science Park, Sir William Lyons Road, Coventry		Consortium member	
Michael	Steele			Micropathology Ltd, The Venture Center, University of Warwick Science Park, Sir William Lyons Road, Coventry		Consortium member	
Jennifer	Holden			Micropathology Ltd, The Venture Center, University of Warwick Science Park, Sir William Lyons Road, Coventry		Consortium member	

*First name, last name, and suffix (if applicable) are required and will appear in PubMed.

***First Name and Middle Initial(s)**	***Last Name**	***Suffix (eg, Jr, III)**	Academic Degrees	Institution	Location (city, state/province, country)	Role or Contribution, eg, chair, principal investigator	Group (if more than 1 Group listed in the byline) and/or Subgroup (eg, Steering Committee)
Andrew	Taylor			Micropathology Ltd, The Venture Center, University of Warwick Science Park, Sir William Lyons Road, Coventry		Consortium member	
Ronan	Calvez			Micropathology Ltd, The Venture Center, University of Warwick Science Park, Sir William Lyons Road, Coventry		Consortium member	
Catherine	Davies			Micropathology Ltd, The Venture Center, University of Warwick Science Park, Sir William Lyons Road, Coventry		Consortium member	
Benjamin	Evans			Micropathology Ltd, The Venture Center, University of Warwick Science Park, Sir William Lyons Road, Coventry		Consortium member	
Jake	Stevens			Micropathology Ltd, The Venture Center, University of Warwick Science Park, Sir William Lyons Road, Coventry		Consortium member	
Peter	Matthews			Micropathology Ltd, The Venture Center, University of Warwick Science Park, Sir William Lyons Road, Coventry		Consortium member	
Kyle	Billing			Micropathology Ltd, The Venture Center, University of Warwick Science Park, Sir William Lyons Road, Coventry		Consortium member	
Werner	Zenz			Medical University of Graz, Austria		Consortium member	
Alexander	Binder			Medical University of Graz, Austria		Consortium member	
Benno	Kohlmaier			Medical University of Graz, Austria		Consortium member	
Daniela S	Kohlfürst			Medical University of Graz, Austria		Consortium member	
Nina A	Schweintzger			Medical University of Graz, Austria		Consortium member	
Christoph	Zurl			Medical University of Graz, Austria		Consortium member	
Susanne	Hösele			Medical University of Graz, Austria		Consortium member	
Manuel	Leitner			Medical University of Graz, Austria		Consortium member	

Supplemental Online Content: Nonauthor Collaborators
*First name, last name, and suffix (if applicable) are required and will appear in PubMed.

***First Name and Middle Initial(s)**	***Last Name**	***Suffix (eg, Jr, III)**	Academic Degrees	Institution	Location (city, state/province, country)	Role or Contribution, eg, chair, principal investigator	Group (if more than 1 Group listed in the byline) and/or Subgroup (eg, Steering Committee)
Lena	Pölz			Medical University of Graz, Austria		Consortium member	
Alexandra	Rusu			Medical University of Graz, Austria		Consortium member	
Glorija	Rajic			Medical University of Graz, Austria		Consortium member	
Bianca	Stoiser			Medical University of Graz, Austria		Consortium member	
Martina	Strempfl			Medical University of Graz, Austria		Consortium member	
Manfred G	Sagmeister			Medical University of Graz, Austria		Consortium member	
Sebastian	Bauchinger			Medical University of Graz, Austria		Consortium member	
Martin	Benesch			Medical University of Graz, Austria		Consortium member	
Astrid	Ceolotto			Medical University of Graz, Austria		Consortium member	
Ernst	Eber			Medical University of Graz, Austria		Consortium member	
Siegfried	Gallistl			Medical University of Graz, Austria		Consortium member	
Harald	Haidl			Medical University of Graz, Austria		Consortium member	
Almuthe	Hauer			Medical University of Graz, Austria		Consortium member	
Christa	Hude			Medical University of Graz, Austria		Consortium member	
Andreas	Kapper			Medical University of Graz, Austria		Consortium member	
Markus	Keldorfer			Medical University of Graz, Austria		Consortium member	
Sabine	Löffler			Medical University of Graz, Austria		Consortium member	
Tobias	Niedrist			Medical University of Graz, Austria		Consortium member	
Heidemarie	Pilch			Medical University of Graz, Austria		Consortium member	
Andreas	Pfleger			Medical University of Graz, Austria		Consortium member	
Klaus	Pfurtscheller			Medical University of Graz, Austria		Consortium member	
Siegfried	Rödl			Medical University of Graz, Austria		Consortium member	
Andrea	Skrabl-Baumgartner			Medical University of Graz, Austria		Consortium member	
Volker	Strenger			Medical University of Graz, Austria		Consortium member	
Elmar	Wallner			Medical University of Graz, Austria		Consortium member	
Maike K	Tauchert			Biobanking and BioMolecular Resources Research Infrastructure - European Research Infrastructure Consortium (BBMRI-ERIC), Neue Stiftingtalstrasse 2/B/6, 8010, Graz, Austria		Consortium member	
Shunmay	Yeung			London School of Hygiene and Tropical Medicine		Consortium member	
Manuel	Dewez			London School of Hygiene and Tropical Medicine		Consortium member	

Supplemental Online Content: Nonauthor Collaborators
*First name, last name, and suffix (if applicable) are required and will appear in PubMed.

*First Name and Middle Initial(s)	*Last Name	*Suffix (eg, Jr, III)	Academic Degrees	Institution	Location (city, state/province, country)	Role or Contribution, eg, chair, principal investigator	Group (if more than 1 Group listed in the byline) and/or Subgroup (eg, Steering Committee)
David	Bath			London School of Hygiene and Tropical Medicine		Consortium member	
Elizabeth	Fitchett			London School of Hygiene and Tropical Medicine		Consortium member	
Fiona	Cresswell			London School of Hygiene and Tropical Medicine		Consortium member	
Effua	Usuf			Medical Research Council Unit The Gambia at LSHTM		Consortium member	
Kalifa	Bojang			Medical Research Council Unit The Gambia at LSHTM		Consortium member	
Anna	Roca			Medical Research Council Unit The Gambia at LSHTM		Consortium member	
Isatou	Sarr			Medical Research Council Unit The Gambia at LSHTM		Consortium member	
Momodou	Saidykhan			Medical Research Council Unit The Gambia at LSHTM		Consortium member	
Ebrahim	Ndure			Medical Research Council Unit The Gambia at LSHTM		Consortium member	
Ulrich	von Both			Dr. von Hauner Children's Hospital, University Hospital, LMU Munich		Consortium member	
Laura	Kolberg			Dr. von Hauner Children's Hospital, University Hospital, LMU Munich		Consortium member	
Patricia	Schmied			Dr. von Hauner Children's Hospital, University Hospital, LMU Munich		Consortium member	
Ioanna	Mavridi			Dr. von Hauner Children's Hospital, University Hospital, LMU Munich		Consortium member	
Irene	Alba-Alejandre			University Hospital, LMU Munich, Munich, Germany		Consortium member	
Nikolaus	Haas			University Hospital, LMU Munich,		Consortium member	
Esther	Maier			Dr. von Hauner Children's Hospital, University Hospital, LMU Munich		Consortium member	
Sabrina	Juranek			Dr. von Hauner Children's Hospital, University Hospital, LMU Munich		Consortium member	
Tobias	Feuchtinger			Dr. von Hauner Children's Hospital, University Hospital, LMU Munich		Consortium member	

Supplemental Online Content: Nonauthor Collaborators
*First name, last name, and suffix (if applicable) are required and will appear in PubMed.

***First Name and Middle Initial(s)**	***Last Name**	***Suffix (eg, Jr, III)**	Academic Degrees	Institution	Location (city, state/province, country)	Role or Contribution, eg, chair, principal investigator	Group (if more than 1 Group listed in the byline) and/or Subgroup (eg, Steering Committee)
Katharina	Danhauser			Dr. von Hauner Children's Hospital, University Hospital, LMU Munich		Consortium member	
Matthias	Griese			Dr. von Hauner Children's Hospital, University Hospital, LMU Munich		Consortium member	
Matthias	Kappler			Dr. von Hauner Children's Hospital, University Hospital, LMU Munich		Consortium member	
Eberhard	Lurz			Dr. von Hauner Children's Hospital, University Hospital, LMU Munich		Consortium member	
Sebastian	Schroepf			Dr. von Hauner Children's Hospital, University Hospital, LMU Munich		Consortium member	
Florian	Hoffmann			Dr. von Hauner Children's Hospital, University Hospital, LMU Munich		Consortium member	
Karl	Reiter			Dr. von Hauner Children's Hospital, University Hospital, LMU Munich		Consortium member	
Carola	Schoen			Dr. von Hauner Children's Hospital, University Hospital, LMU Munich		Consortium member	
Philipp	Agyeman			Bern University Hospital, University of Bern, Switzerland		Consortium member	
Christoph	Aebi			Bern University Hospital, University of Bern, Switzerland		Consortium member	
Luregn J	Schlapbach			University Children`s Hospital Zurich		Consortium member	
Eric	Giannoni			Lausanne University Hospital and University of Lausanne, Switzerland		Consortium member	
Martin	Stocker			Children's Hospital Lucerne, Switzerland		Consortium member	
Klara M	Posfay-Barbe			Children's Hospital of Geneva, University Hospitals of Geneva, Switzerland		Consortium member	
Ulrich	Heininger			University of Basel Children's Hospital, Switzerland		Consortium member	
Sara	Bernhard-Stirnemann			Children's Hospital Aarau, Switzerland		Consortium member	
Anita	Niederer-Loher			Children's Hospital of Eastern Switzerland St. Gallen, Switzerland		Consortium member	
Christian	Kahlert			Children's Hospital of Eastern Switzerland St. Gallen, Switzerland		Consortium member	

Supplemental Online Content: Nonauthor Collaborators
*First name, last name, and suffix (if applicable) are required and will appear in PubMed.

***First Name and Middle Initial(s)**	***Last Name**	***Suffix (eg, Jr, III)**	Academic Degrees	Institution	Location (city, state/province, country)	Role or Contribution, eg, chair, principal investigator	Group (if more than 1 Group listed in the byline) and/or Subgroup (eg, Steering Committee)
Giancarlo	Natalucci			University Hospital Zurich, Switzerland		Consortium member	
Christa	Relly			Children's Research Center, University Children's Hospital Zurich, Switzerland		Consortium member	
Christoph	Berger			Children's Research Center, University Children's Hospital Zurich, Switzerland		Consortium member	
Thomas	Riedel			Children's Hospital Chur, Switzerland		Consortium member	
