# Supplementary material for: Evaluation of Autoantibody Binding to Cardiac Tissue in Multisystem Inflammatory Syndrome in Children and COVID-19 Vaccination–Induced Myocarditis
Source: JAMA Netw Open. 2023 May 18;6(5):e2314291. doi: 10.1001/jamanetworkopen.2023.14291 (PMC10196878; doi:10.1001/jamanetworkopen.2023.14291)
Supplement: Supplement 3. — Data Sharing Statement [file jamanetwopen-e2314291-s003.pdf]

Data Sharing Statement

Patel. Evaluation of Autoantibody Binding to Cardiac Tissue in Multisystem Inflammatory Syndrome in Children and COVID-19 Vaccination–Induced Myocarditis. *JAMA Netw Open*. Published May 18, 2023. doi:10.1001/jamanetworkopen.2023.14291

Data
Data available: Yes
Data types: Deidentified participant data
How to access data: harsita.patel08@imperial.ac.uk
When available: With publication

Supporting Documents
Document types: None

Additional Information
Who can access the data: researchers whose proposed use of the data has been approved
Types of analyses: for a specified purpose
Mechanisms of data availability: with a signed data access agreement
